# Transient HIF2A inhibition promotes satellite cell proliferation and muscle regeneration

**DOI:** 10.1172/JCI96208

**Published:** 2018-04-30

**Authors:** Liwei Xie, Amelia Yin, Anna S. Nichenko, Aaron M. Beedle, Jarrod A. Call, Hang Yin

**Affiliations:** 1 Department of Biochemistry and Molecular Biology,; 2 Center for Molecular Medicine, and; 3 Department of Kinesiology, The University of Georgia, Athens, Georgia, USA.; 4 Department of Pharmaceutical Sciences, Binghamton University-SUNY, Binghamton, New York, USA.; 5 Regenerative Bioscience Center, The University of Georgia, Athens, Georgia, USA.

**Keywords:** Muscle Biology, Stem cells, Adult stem cells, Skeletal muscle

## Abstract

The remarkable regeneration capability of skeletal muscle depends on the coordinated proliferation and differentiation of satellite cells (SCs). The self-renewal of SCs is critical for long-term maintenance of muscle regeneration potential. Hypoxia profoundly affects the proliferation, differentiation, and self-renewal of cultured myoblasts. However, the physiological relevance of hypoxia and hypoxia signaling in SCs in vivo remains largely unknown. Here, we demonstrate that SCs are in an intrinsic hypoxic state in vivo and express hypoxia-inducible factor 2A (HIF2A). HIF2A promotes the stemness and long-term homeostatic maintenance of SCs by maintaining their quiescence, increasing their self-renewal, and blocking their myogenic differentiation. HIF2A stabilization in SCs cultured under normoxia augments their engraftment potential in regenerative muscle. Conversely, HIF2A ablation leads to the depletion of SCs and their consequent regenerative failure in the long-term. In contrast, transient pharmacological inhibition of HIF2A accelerates muscle regeneration by increasing SC proliferation and differentiation. Mechanistically, HIF2A induces the quiescence and self-renewal of SCs by binding the promoter of the *Spry1* gene and activating *Spry1* expression. These findings suggest that HIF2A is a pivotal mediator of hypoxia signaling in SCs and may be therapeutically targeted to improve muscle regeneration.

## Introduction

The coordinated proliferation and differentiation of adult stem cells provide sustainable cell sources for tissue repair and regeneration. In support of long-term tissue homeostasis, many adult stem cell populations fine-tune the balance between proliferative and quiescent states so as to both meet the need for tissue repair and lessen cell cycle–associated stresses. Ineffective reversal of proliferation and quiescence in adult stem cells has been implicated in aging and many diseases (1).

Adult skeletal muscle stem cells, also called satellite cells (SCs), are essential for skeletal muscle regeneration (2). SCs and their undifferentiated progeny universally express paired box 7 (Pax7), a transcription factor that has been utilized to identify both quiescent and proliferative SCs or manipulate gene expression specifically in SCs (3, 4). In uninjured muscle, SCs reside closely juxtaposed with contractile myofibers beneath the basal lamina and are mitotically quiescent. SC quiescence is jointly maintained by, but not limited to, multiple mechanisms including TTP-mediated RNA decay, SIRT1-dependent histone deacetylation, and restraint of myogenic and proliferation-associated gene expression by microRNAs (5–8). Upon muscle injury, SCs transit through a G_(Alert)_ phase and enter the cell cycle in response to the activation of HGF/FGF-mediated RTK signaling and p38/MAPK signaling (9–11). After limited rounds of proliferation, a subset of SCs returns to quiescence and the niche location adjacent to myofibers (self-renewal), whereas many SCs undergo myogenic differentiation and eventually repair damaged myofibers. Angiopoietin-1/Tie2 RTK signaling, the RTK negative regulator Spry1, and inactivation of p38/MAPK signaling promote SC self-renewal (11–13). Besides these intrinsic mechanisms, dynamic remodeling of the muscle microenvironment also plays a critical role in directing SC behavior during muscle repair (2). However, how SCs fine-tune the scale of reversible quiescence and balance proliferation versus differentiation remains incompletely understood.

Oxygen (O_2_) is essential for mitochondrial bioenergetics and energy homeostasis; low O_2_ tension (hypoxia) underlies many disease conditions (14). The cellular responses to hypoxia are mainly mediated by hypoxia-inducible factors (HIFs), which form heterodimeric complexes between an O_2_-sensitive α-subunit (HIF1A/HIF2A/HIF3A) and a stable β-subunit (HIF1B) (15). Under normoxia, the α-subunit of HIF is hydroxylated and targeted for proteasome degradation. Under hypoxia, limited O_2_ stabilizes the HIF complex, which binds hypoxia-responsive elements (HREs) (5′-RCGTG-3′) and activates target gene expression. Many types of adult stem cells prefer hypoxic niches to maintain their quiescent and/or undifferentiated states (16). In vitro hypoxic conditions also profoundly impact the proliferation and differentiation of cultured SCs (myoblasts) (17). In addition, HIF1A KO or compound HIF1A/HIF2A double-KO in adult SCs distinctly affects SC numbers and muscle regeneration (18, 19). Yet, it remains elusive whether hypoxia is of any physiological relevance to SC in vivo and, if so, whether HIF-mediated hypoxia signaling regulates SC quiescence or can be potentially targeted to improve muscle regeneration.

Here, we demonstrated that SCs are inherently hypoxic within their in vivo niche. Quiescent SCs (QSCs) expressed HIF2A, but not HIF1A. We found that HIF2A was essential for the maintenance of SCs in a quiescent and undifferentiated state and for vff

## Results

### QSCs are hypoxic in the niche and express HIF2A, but not HIF1A.

**Figure 1:**
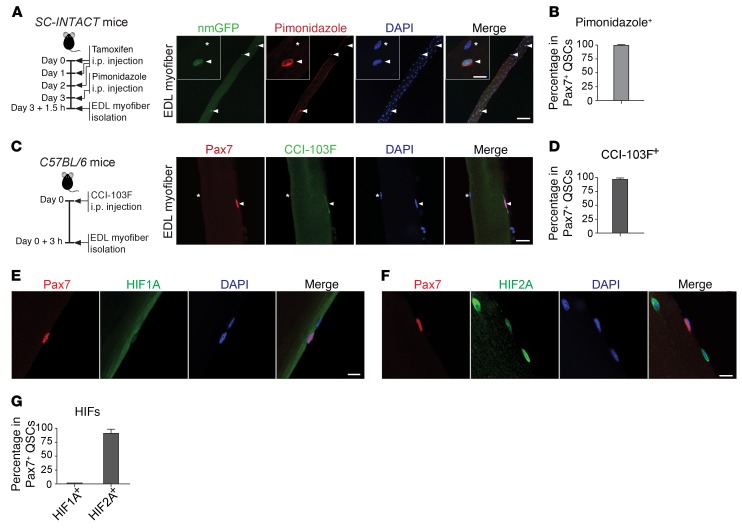
QSCs are hypoxic in the niche and express HIF2A, but not HIF1A. (**A**) Timeline of in vivo pimonidazole labeling in *SC-INTACT* mice and representative confocal images of uninjured/resting EDL myofibers (*n* >50 myofibers from *n* = 3 mice) showing that nmGFP*^+^* QSCs were pimonidazole^+^. Scale bars: 50 μm and 10 μm (insets). Inset images show that pimonidazole signals were relatively enriched in the cytoplasm of QSCs. Arrowheads indicate a QSC; asterisks indicate a myonucleus. (**B**) Percentage of pimonidazole^+^ QSCs. (**C**) Timeline of in vivo CCI-103F labeling in *C57BL/6* mice and representative images of uninjured/resting EDL myofibers (*n* >50 myofibers from 3 mice) showing that nmGFP*^+^* QSCs were CCI-103F^+^. Arrowheads indicate a QSC; asterisks indicate a myonucleus. Scale bar: 20 μm. (**D**) Percentage of CCI-103F^+^ QSCs. (**E** and **F**) Representative images of uninjured/resting EDL myofibers from *C57BL/6* mice (*n* >50 myofibers from 6 mice/group) showing that most QSCs were HIF2A^+^, but HIF1A^–^. Scale bars: 10 μm. (**G**) Percentage of HIF1A^+^ and HIF2A^+^ QSCs. Data represent the mean ± SEM.

The partial oxygen tension (pO_2_) of most adult tissues ranges from approximately 2% to 9% (20). To assess the intracellular pO_2_ of SCs in their niche, a hypoxia probe, pimonidazole, was administered in vivo. This probe forms protein adducts in hypoxic cells in situ (where pO_2_ is <1.3%) (21). Because the antibodies against the SC marker Pax7 and pimonidazole are of the same isotype, pimonidazole was i.p. injected into tamoxifen-treated 3-month-old adult *Pax7^Cre/ERT2^ R26R^CAG-Sun1/sfGFP^* mice (hereafter referred to as *SC-INTACT* mice), within which greater than 99% of Pax7^+^ QSCs were genetically labeled with nuclear membrane–located GFP (nmGFP) in the muscle (Supplemental Figure 1, A–C; supplemental material available online with this article; https://doi.org/10.1172/JCI96208DS1) (22). On myofibers isolated from extensor digitorum longus (EDL) muscles, we detected abundant pimonidazole adducts in over 99% of nmGFP^+^ QSCs (particularly within the cytoplasm), indicating that QSCs were in a hypoxic state in vivo (Figure 1, A and B). We further confirmed the hypoxic state of QSCs using another hypoxia probe, CCI-103F. In adult WT *C57BL/6* mice, we detected CCI-103F adducts in the cytoplasm of approximately 97% of Pax7^+^ QSCs (Figure 1, C and D). In contrast to SCs, the sarcoplasm of myofibers or myonuclei had little staining for pimonidazole/CCI-103F. Given this clear contrast, we investigated whether pimonidazole is capable of detecting hypoxia within myofibers. EDL myofibers from *SC-INTACT* mice were cultured ex vivo under hypoxic conditions (1% pO_2_) and labeled with pimonidazole in the culture media (Supplemental Figure 1D). Under this defined hypoxic condition, we detected pimonidazole adducts in both the cytoplasm and nuclei of SCs, in the sarcoplasm of myofibers, and at the periphery of myonuclei, but not within myonuclei (Supplemental Figure 1E). Thus, QSCs were hypoxic in vivo, whereas the adjacent myofibers were likely not hypoxic. The nonhypoxic state of myofibers is consistent with the notion that the physiological pO_2_ of resting skeletal muscle is approximately 4.2% (23, 24).

Our previous RNA-sequencing (RNA-seq) analysis revealed abundant levels of HIF1A and HIF2A mRNA, yet no HIF3A transcripts in QSCs (25). To understand whether HIFs are stabilized in hypoxic QSCs, we stained for HIF1A and HIF2A in uninjured resting muscles from 3-month-old adult *C57BL/6* mice. We did not detect HIF1A in most Pax7^+^ QSCs (Figure 1, E and G, and Supplemental Figure 1F), whereas approximately 90% of QSCs had clear nuclear staining for HIF2A (Figure 1, F and G, and Supplemental Figure 1G). Given that hypoxia-stabilized HIFs function as transcription factors in the nucleus, the above observations indicate that HIF2A, not HIF1A, is selectively stabilized and expressed in hypoxic QSCs.

### Muscle repair following eccentric contraction–induced injury is concomitant with dynamic alterations of HIF2A and HIF1A expression in SCs.

**Figure 2:**
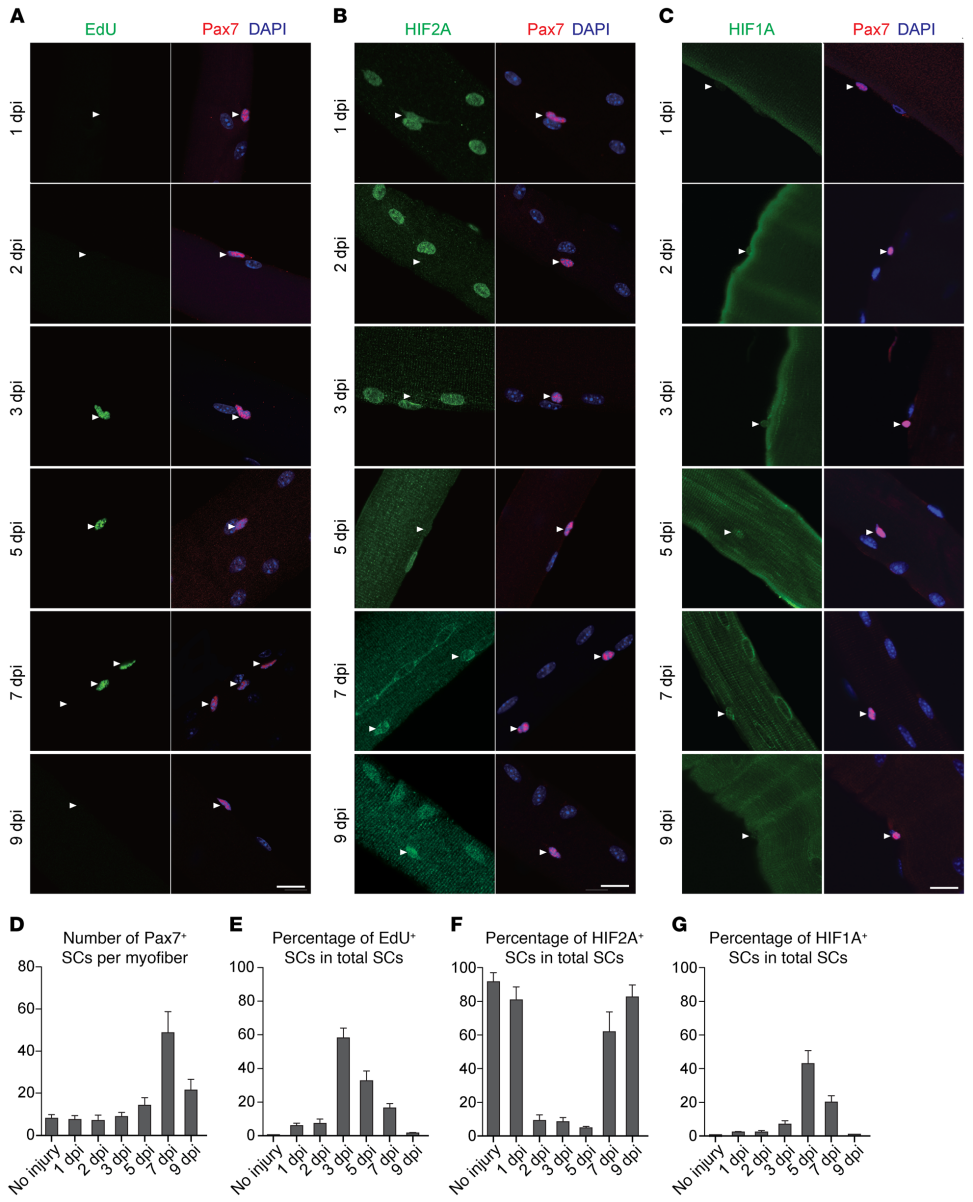
Muscle repair following eccentric contraction–induced injury is concomitant with dynamic alterations of HIF2A and HIF1A expression in SCs. (**A**–**C**) Representative images of EDL myofibers from injured muscles at various time points (*n* >50 myofibers from 3 mice/group/time point) and stained for Pax7, DAPI, and EdU (**A**), HIF2A (**B**), or HIF1A (**C**). Scale bars: 20 μm. Arrowheads indicate SCs. (**D**) Number of Pax7^+^ SCs per myofiber at various time points. (**E**) Percentage of EdU^+^ SCs at various time points. (**F**) Percentage of HIF2A^+^ SCs at various time points. (**G**) Percentage of HIF1A^+^ SCs at various time points. Data represent the mean ± SEM.

To assess the expression of HIFs in proliferative SCs during a physiological process of muscle repair, tibialis anterior (TA) and EDL muscles of adult *C57BL/6* mice were injured by eccentric contraction, and the injured EDL myofibers were isolated from 1 to 9 days post injury (dpi) (Supplemental Figure 2A). EdU and Evans blue were administered 24 hours before each myofiber isolation to assess cell proliferation and sarcolemma integrity, respectively. After the stretch-induced injury, Pax7^+^ SCs on injured EDL myofibers increased by 5 dpi, reached a peak on 7 dpi, and declined from 7 to 9 dpi (Figure 2D), which correlates with the repair process as evidenced by the progressive restoration of sarcolemma integrity (Supplemental Figure 2B). The dynamics of SC numbers also correlated with the proliferative state of SCs (percentages of EdU^+^ SCs), which slightly increased at 1 to 2 dpi, peaked at 3 dpi, and decreased from 3 to 9 dpi (Figure 2, A and E). The expression of HIF2A in SCs was altered during the repair process: at 1 dpi, approximately 80% of SCs remained HIF2A^+^; from 2 to 5 dpi, only less than 10% of SCs were HIF2A^+^; and HIF2A reappeared in approximately 60% of SCs at 7 dpi and was expressed in approximately 83% of SCs by 9 dpi (Figure 2, B and F). The dynamics of HIF2A expression and SC proliferation showed opposite trends (Pearson’s correlation coefficient = –0.71).

As HIF2A diminished in SCs from 1 to 2 dpi, we investigated whether hypoxia subsided in SCs at the same time. Pimonidazole was administered in stretch-injured *SC-INTACT* mice at 1, 2, and 3 dpi, when more than 90% of nmGFP^+^ cells on injured EDL myofibers were still Pax7^+^ and remained as undifferentiated SCs (data not shown). At 1 to 3 dpi, we observed that pimonidazole was abundant in most nmGFP^+^ SCs, including those HIF2A^–^ SCs (at 2 and 3 dpi), indicating that SCs remained hypoxic when HIF2A diminished (Supplemental Figure 2, C and D).

The sustained hypoxia in SCs might be permissive for HIF1A stabilization. Indeed, we detected discernible yet weak HIF1A staining in approximately 7% of Pax7^+^ SCs at 3 dpi, in approximately 43% of SCs at 5 dpi, and in approximately 20% of SCs at 7 dpi, but was undetectable at 9 dpi (Figure 2, C and G). The dynamics of HIF1A expression in SCs appeared to be positively correlated with SC proliferation (Pearson’s correlation coefficient = +0.42), corroborating the reported function of HIF1A in promoting SC proliferation under hypoxia (26).

### Genetic ablation of HIF2A in QSCs leads to transient activation, proliferation, and differentiation of SCs.

**Figure 3:**
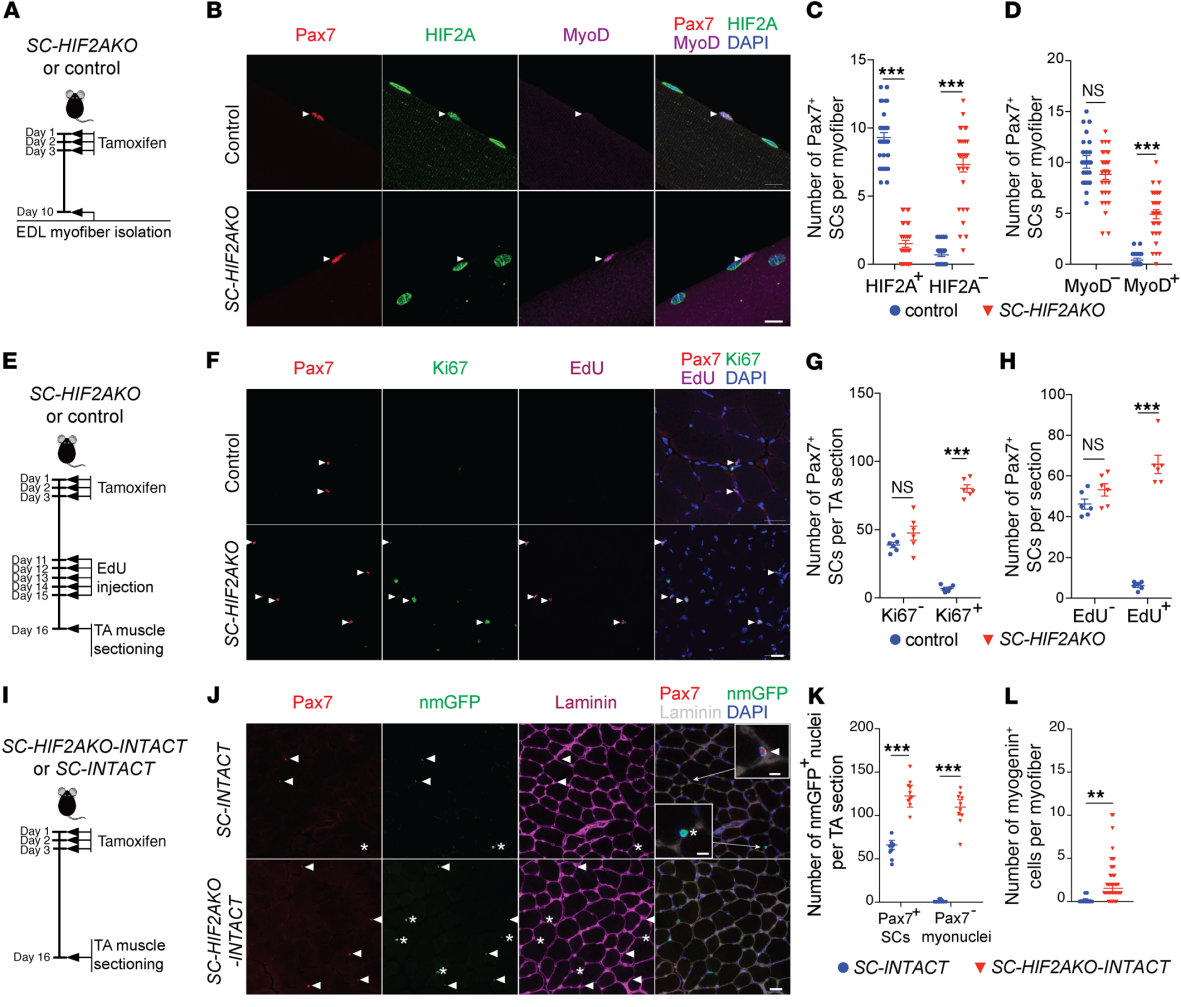
Genetic ablation of HIF2A in QSCs leads to transient activation, proliferation, and differentiation of SCs. (**A**) Timeline of genetic ablation of HIF2A in QSCs. (**B**) Representative images of myofibers from *SC-HIF2AKO* mice and control littermates (*n* >50 myofibers from 5 mice/group; 10 dpr). Immunofluorescence of Pax7 (red), HIF2A (green), MyoD (purple), and DAPI (blue) staining revealed HIF2A^–^MyoD^+^ and HIF2A^+^MyoD^–^ SCs (arrowheads) in *SC-HIF2AKO* and control mice, respectively. Scale bar: 10 μm. (**C**) Number of HIF2A^+^ and HIF2A^–^ SCs per myofiber (10 dpr). (**D**) Number of MyoD^–^ and MyoD^+^ SCs per myofiber (10 dpr). (**E**) Timeline characterizing SC proliferation after HIF2A ablation in QSCs. (**F**) Representative cross-sectional images of TA muscles from *SC-HIF2AKO* mice and control littermates (*n* = 6 mice/group; 16 dpr). Immunofluorescence of Pax7 (red), Ki67 (green), EdU (purple), and DAPI (blue) staining revealed an increase in Ki67^+^EdU^+^ SCs (arrowheads) in *SC-HIF2AKO* mice. Scale bar: 20 μm. (**G**) Number of Ki67^–^ and Ki67^+^ SCs per TA section. (**H**) Number of EdU^–^ and EdU^+^ SCs per TA section. (**I**) Timeline for tracing SC fates after HIF2A ablation in QSCs. (**J**) Representative images of TA muscles from *SC-HIF2AKO-INTACT* and control *SC-INTACT* mice (*n* = 6 mice/group; 16 dpr). Immunofluorescence of nmGFP, Pax7, laminin B2, and DAPI revealed increased nmGFP^+^Pax7^+^ SCs (arrowheads) and nmGFP^+^Pax7^–^ myonuclei (asterisks) in *SC-HIF2AKO-INTACT* mice. Scale bar: 20 μm and 5 μm (insets). Inset images show that both nmGFP^+^Pax7^+^ SCs and nmGFP^+^Pax7^–^ myonuclei are adjacent to the basal lamina. (**K**) Number of nmGFP^+^Pax7^+^ SCs and nmGFP^+^Pax7^–^ myonuclei per TA section. (**L**) Number of nmGFP^+^myogenin^+^ differentiating SCs per EDL myofiber (16 dpr). **P < 0.01 and ***P < 0.005, by 2-sided Student’s *t* test. Data represent the mean ± SEM.

Upon muscle injury, the robust SC proliferation following diminished HIF2A expression implicates a role for HIF2A in the maintenance of SC quiescence. To investigate this potential function of HIF2A, we administered tamoxifen to 3-month-old adult *Pax7^cre/ERT2^ HIF2A^fl/fl^* mice (hereafter referred to as *SC-HIF2AKO* mice), which resulted in genetic ablation of HIF2A specifically in SCs (Figure 3, A and B). At 10 days post tamoxifen-induced recombination (10 dpr), we found that HIF2A expression was abolished in approximately 80% of SCs in uninjured EDL myofibers (Figure 3, B and C). The myogenic transcription factor MyoD is absent in QSCs yet expressed in activated/proliferative SCs (27). We found that HIF2A ablation led to a remarkable increase in MyoD^+^ SCs in uninjured myofibers (Figure 3, B and D), suggesting that HIF2A-depleted SCs were spontaneously activated in uninjured muscles.

To confirm that SCs proliferate after HIF2A ablation, we administered EdU to *SC-HIF2AKO* mice at 11 to 15 dpr (Figure 3E). At 16 dpr, uninjured TA muscles from *SC-HIF2AKO* mice contained increased numbers of Pax7^+^Ki67^+^ SCs and Pax7^+^EdU^+^ SCs compared with those from control mice (Figure 3, F–H). We detected the proliferation markers Ki67 and EdU only in Pax7^+^ SCs, consistent with the specificity of HIF2A ablation in the SC compartment (Figure 3F).

To investigate whether SCs differentiate following proliferation, we performed SC lineage tracing in *Pax7^cre/ERT2^* *HIF2A^fl/fl^* *R26R^CAG-Sun1/sfGFP^* mice (hereafter referred to as *SC-HIF2AKO-INTACT* mice) and control *SC-INTACT* mice, in which nmGFP-labeled SCs may remain as Pax7^+^ SCs or differentiate into Pax7^–^ myonuclei (Figure 3, I and J). At 16 dpr, we observed that nmGFP^+^Pax7^+^ SCs increased in uninjured TA muscles from *SC-HIF2AKO-INTACT* mice, confirming SC proliferation upon HIF2A ablation; meanwhile, these uninjured, resting muscles also contained increased nmGFP^+^Pax7^–^ myonuclei, indicating massive myogenic differentiation of HIF2A-ablated SCs (Figure 3, J and K). The myogenic transcription factor myogenin is specifically expressed in differentiating myogenic cells (2). Uninjured EDL myofibers from *SC-HIF2AKO-INTACT* mice (16 dpr) contained an increased number of nmGFP^+^myogenin^+^ cells compared with myofibers from control mice (Figure 3L and Supplemental Figure 3A), further confirming that HIF2A ablation in SCs induces myogenic differentiation.

To investigate whether SC apoptosis follows HIF2A ablation, we performed TUNEL labeling of TA muscle sections from *SC-HIF2AKO-INTACT* and control *SC-INTACT* mice. For both types of mice, TUNEL^+^ apoptotic nuclei counted for less than 0.5% of nmGFP^+^ nuclei (of SCs and SC progeny) at 16 dpr (Supplemental Figure 3, B and C). In contrast, TUNEL^+^ nuclei were readily detected in nuclease-treated muscle sections (technical positive control; Supplemental Figure 3D). Thus, SCs undergo activation, proliferation, and differentiation, but not apoptosis, in uninjured, resting muscles after HIF2A ablation.

It has been previously reported that the depletion of 1 HIF α-subunit provokes an increase of the other (28). At 16 dpr, we detected weak HIF1A expression in approximately 15% of Pax7^+^ SCs on uninjured EDL myofibers from *SC-HIF2AKO* mice, but not in SCs from control EDL myofibers (Supplemental Figure 3, E and F). As HIF2A expression was abolished in more than 90% of SCs at 16 dpr (data not shown), HIF2A depletion appeared not to induce HIF1A compensation in the majority of SCs.

### Long-term ablation of HIF2A results in the loss of SC homeostatic self-renewal.

**Figure 4:**
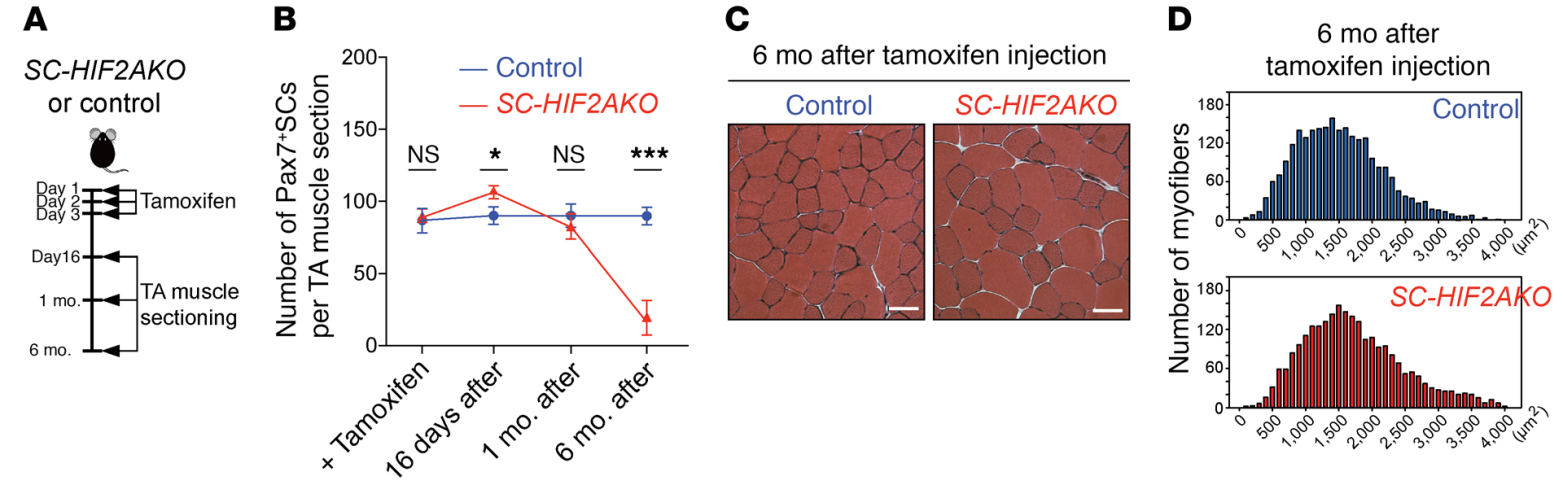
Long-term ablation of HIF2A results in the loss of SC homeostatic self-renewal. (**A**) Timeline characterizing SC homeostasis after HIF2A ablation in QSCs. (**B**) Number of Pax7^+^ SCs per TA section in *SC-HIF2AKO* mice and control littermates on the same day of tamoxifen induction (+ Tamoxifen), 16 days, 1 month, and 6 months after tamoxifen-induced HIF2A ablation (*n* = 3 mice/group/time point). *P < 0.05 and ***P < 0.005, by 2-sided Student’s *t* test. Data represent the mean ± SEM. (**C**) H&E staining of TA muscles from *SC-HIF2AKO* mice and control littermates (*n* = 3 mice/group; 6 mo after tamoxifen-induced HIF2A ablation). Scar bars: 20 μm. (**D**) Distribution of myofiber cross-sectional areas of TA muscles from *SC-HIF2AKO* mice and control littermates (*n* = 3 mice/group; 6 mo after tamoxifen-induced HIF2A ablation).

The long-lasting regeneration capability of skeletal muscle depends on the self-renewal and homeostatic maintenance of SCs. We next investigated the impact of HIF2A ablation on SC self-renewal. Upon tamoxifen-induced HIF2A ablation, the number of Pax7^+^ SCs in TA muscles from *SC-HIF2AKO* mice increased to approximately 110% by day 16, returned to normal levels at 1 month, and fell to approximately 20% of normal levels at 6 months (Figure 4, A and B), revealing that HIF2A ablation impaired SC self-renewal and homeostatic maintenance. Six months after tamoxifen treatment, SC-depleted muscles in *SC-HIF2AKO* mice showed a discernible increase in myofiber calibers compared with muscles from age-matched controls (Figure 4, C and D), yet with comparable maximal torques (Supplemental Figure 3G). Therefore, HIF2A is essential for the quiescence and long-term homeostatic maintenance of SCs in adult uninjured muscle.

### HIF2A stabilization under normoxia promotes quiescence, self-renewal, and stemness of SCs yet impedes myogenic differentiation.

Hypoxia treatment increases the engraftment efficiency of cultured SCs (myoblasts) yet impairs their proliferation and differentiation (29, 30). The above phenotypes of HIF2A-ablated SCs led us to investigate whether HIF2A stabilization under normoxia is sufficient to elicit effects similar to those of hypoxia, which would be beneficial to SC transplantation–based therapy. After 72 hours in normoxic culture (21% pO_2_), SC clusters formed on myofibers as a result of the activation and proliferation of individual SCs. Unlike the nuclear localization of Pax7 and MyoD, we detected HIF2A in the cytoplasm of SCs, suggestive of its loss of function under normoxia (Supplemental Figure 4A). The cytoplasmic staining was specific to HIF2A, as evidenced by its absence in SC clusters derived from HIF2A-ablated SCs (16 dpr; Supplemental Figure 4B). Like cultured SCs, C2C12 myoblasts cultured under normoxia also expressed a low but detectable level of HIF2A that was mainly present in the cytoplasmic fraction, which is in line with the notion that normoxia destabilizes HIF2A (Supplemental Figure 4C).

**Figure 5:**
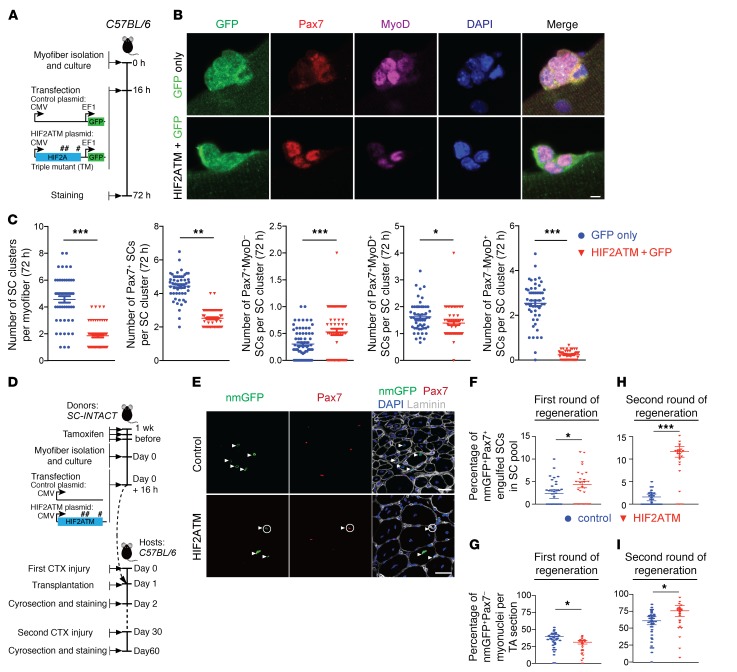
HIF2A stabilization under normoxia promotes quiescence, self-renewal, and stemness of SCs yet impedes myogenic differentiation. (**A**) Diagram depicting the timeline and plasmids used for HIF2A stabilization in normoxic SC culture. Pound signs denote the number and locations of point mutations in HIF2ATM. (**B**) Representative images of transfected (GFP^+^) SC clusters on myofibers from *C57BL/6* mice (*n* >50 myofibers from 7 mice/group). The SC clusters were transfected with either HIF2ATM or control plasmids and stained for Pax7, MyoD, and DAPI. Scale bar: 5 μm. (**C**) Number of SC clusters, Pax7^+^ SCs per SC cluster, and Pax7^+^MyoD^–^, Pax7^+^MyoD^+^, and Pax7^–^MyoD^+^ SCs per SC cluster (*n* >50 myofibers). (**D**) Diagram of the experimental scheme for transplantation of HIF2A-stabilized SCs and tracing of their cell fates in vivo. (**E**) Cross-sectional images of TA muscles that were transplanted with HIF2ATM- or control plasmid–transfected SCs (*n* = 5 mice/group; 21 days after the first injury). Immunofluorescence of Pax7 and nmGFP revealed 2 fates of transplanted SCs: engrafted SCs that retained stemness (nmGFP^+^Pax7^+^; circles, bottom) and engrafted SCs that differentiated into myonuclei (nmGFP^+^Pax7^–^; arrowheads). Scale bars: 20 μm. (**F** and **H**) Percentage of engrafted and self-renewed nmGFP^+^Pax7^+^ SCs in the total SC pool after the first round (21 dpi; *n* = 5 mice/group in **F**) and second round (30 dpi; *n* = 6 mice/group in **H**) of regeneration. (**G** and **I**) Number of nmGFP^+^Pax7^–^ differentiated myonuclei per TA muscle section after the first round (**G**) and second round (**I**) of regeneration. *P < 0.05, **P < 0.01, and ***P < 0.005, by 2-sided Student’s *t* test. Data represent the mean ± SEM.

To stabilize HIF2A in SCs under normoxia, we transfected cultured SCs with a plasmid expressing GFP (to trace transfection) and a triple mutated form of HIF2A (HIF2ATM, carrying P405A, P530V, and N851A mutations of murine HIF2A), which is resistant to O_2_-induced hydroxylation and proteasome degradation (Figure 5A) (31). After 72 hours of normoxic culture, control myofibers that were transfected only with GFP had 4.6 GFP^+^ SC clusters and 4.3 GFP^+^ SCs per cluster on average (Figure 5, B and C). In contrast, myofibers that were transfected with HIF2ATM-GFP had a markedly reduced number and size of GFP^+^ SC clusters (~1.9 clusters per myofiber and ~2.5 SCs per cluster on average; Figure 5, B and C). Thus, HIF2A stabilization elicited effects opposite those of HIF2A ablation, supporting the idea that HIF2A promotes SC quiescence and impedes SC proliferation. In comparison, the transfection of a WT form of HIF2A (HIF2AWT) under normoxic conditions did not alter the number or size of SC clusters (Supplemental Figure 4D).

As a SC niche factor, myofibers in the above experiment were GFP^+^ and thus probably had altered HIF2A expression. Therefore, we sought to determine whether HIF2A promotes SC quiescence in a cell-autonomous manner. Transfection of HIF2ATM, but not HIF2AWT, into primary myoblasts under normoxia decreased the proliferation rate (Supplemental Figure 4E). Cell-cycle analysis revealed that HIF2ATM transfection increased the percentage of primary myoblasts in G_0_/G_1_ phases and decreased the percentage of cells in G_2_/S phases (Supplemental Figure 4F), indicating that HIF2A stabilization in SCs is intrinsically sufficient to promote quiescence. In contrast to normoxic culture, primary myoblasts proliferated slowly under hypoxia (1% pO_2_), and HIF2ATM transfection did not further repress the proliferation (Supplemental Figure 4E). This observation corroborates with previous findings that hypoxia impairs myoblasts proliferation (17, 29) and implicates a role of HIF2A stabilization in this hypoxic effect.

To assess the impact of HIF2A stabilization on SC self-renewal and differentiation, we stained the normoxia-cultured SC clusters/myofibers for Pax7 and MyoD (Figure 5, A–C). Compared with the control myofibers, HIF2ATM-transfected myofibers had increased numbers of Pax7^+^MyoD^–^ self-renewing SCs (HIF2ATM, 0.6 vs. control, 0.35 cells per cluster) yet decreased numbers of Pax7^–^MyoD^+^ committed myogenic SCs (HIF2ATM, 0.4 vs. control, 2.6 SCs per cluster) (Figure 5C). No such difference was observed after HIF2AWT transfection (Supplemental Figure 4D). Thus, HIF2A stabilization promotes the self-renewal but impairs the myogenic differentiation of SCs.

To confirm that the above effects are SC autonomous, control and HIF2ATM-transfected C2C12 myoblasts were differentiated in 2% horse serum for 5 days (21% pO_2_) and stained for Pax7 and myosin heavy chain (MyHC) (Supplemental Figure 4G). Myoblasts transfected with the control plasmid (GFP^+^) were either Pax7^+^MyHC^–^ (self-renewed reserve cells) or Pax7^–^MyHC^+^ (differentiated myocytes). In contrast, cells transfected with HIF2ATM (GFP^+^) were almost exclusively Pax7^+^MyHC^–^ (Supplemental Figure 4G), confirming that HIF2A intrinsically impedes myogenic differentiation but augments self-renewal.

The above observations suggest that HIF2A stabilization may facilitate the engraftment efficiency of SCs after transplantation (remaining as stem cells). To assess this potential effect, we isolated myofibers (and residing SCs as donor cells) from *SC-INTACT* mice. The specific presence of nmGFP on the SC nuclear membrane allowed us to trace the location and fates of transplanted SCs. We transfected the myofibers with control and HIF2ATM plasmids (no GFP expression) and transplanted them into cardiotoxin-injured (CTX-injured) TA muscles (1 dpi) of cognate 3-month-old *C57BL/6* host mice (Figure 5D). As massive SC transplantation may alter muscle regeneration progression and hence indirectly affects SC fates, we only transplanted 10 myofibers (either control or HIF2A-transfected) into each injured TA muscle. Twenty-one days after transplantation, we found the nmGFP^+^ nuclei to be Pax7^–^ differentiated myonuclei at the center of regenerated myofibers and in niche-residing Pax7^+^ self-renewed SCs (adjacent to the basal lamina), representing two distinct fates of nmGFP^+^ donor SCs (Figure 5E). HIF2ATM transfection increased the number of nmGFP^+^Pax7^+^ engrafted SCs (control, 2.2 SCs vs. HIF2A, 4.5 SCs per section) (Supplemental Figure 4H) and the percentage of engrafted SCs in the total Pax7^+^ SC pool (control, 2.5% vs. HIF2A, 4.8%) (Figure 5F), indicating that HIF2A improves SC engraftment efficiency. In contrast, HIF2ATM transfection decreased the number of nmGFP^+^Pax7^–^ myonuclei (derived from nmGFP^+^ donor SCs; control, 43 nmGFP^+^ myonuclei vs. HIF2A, 32 nmGFP^+^ myonuclei) (Figure 5G), which echoes the negative effect of HIF2A stabilization on myogenic differentiation in cultured SCs and myoblasts.

The long-term outcome of SC transplantation depends on a fine balance between the self-renewal and differentiation of engrafted SCs; either excessive self-renewal or unrestrained differentiation expectedly leads to a failure to support repetitive muscle repair. Thus, we further investigated whether the skewed self-renewal and differentiation potential of HIF2A-stabilized SCs should eventually enhance the long-term engraftment of these stem cells and their contribution to muscle repair. To this aim, we injured the regenerated TA muscles that received SC engraftment (either control or HIF2ATM-transfected SCs) for the second time (Figure 5D). At 30 dpi, the TA muscles from both groups completed regeneration and contained regenerated myofibers of comparable calibers (data not shown). Compared with control TA muscles, the TA muscles that originally engrafted HIF2ATM-transfected SCs contained markedly increased numbers of nmGFP^+^Pax7^+^ donor-originated SCs (control, 2.1 SCs vs. HIF2A, 11.2 engrafted SCs per section) (Supplemental Figure 4I), which was consistent with the results of the first injury and further confirms that HIF2A stabilization improves SC self-renewal. Compared with the first round of regeneration, HIF2ATM-transfected SCs showed a markedly increased proportion in the total Pax7^+^ SC pool after the second regeneration (the first injury, 4.8% vs. the second injury, 11.6%) (Figure 5, F and H). This increase was probably due to superior self-renewal capabilities of HIF2ATM-transfected SCs compared with endogenous SCs and implicates a possible domination of these engrafted SCs in long-term repetitive muscle regeneration. Regarding the myogenic differentiation, the TA muscles that originally engrafted HIF2ATM-transfected SCs also contained increased numbers of nmGFP^+^Pax7^–^ donor–derived myonuclei at the end of the second round of regeneration (control, 61.3 vs. HIF2A, 76.5 nmGFP^+^ myonuclei) (Figure 5I). Thus, although there were twice as many HIF2ATM-transfected SCs as there were control engrafted SCs (an average of 4.5 vs. an average of 2.2 = 200%) at the beginning of the second regeneration, we observed only a moderate increase in donor-derived myonuclei (76.5/61.3 = 124%). This likely reflects the negative impact of HIF2A stabilization on SC proliferation and/or differentiation. However, the overall enhancement of the contribution to muscle regeneration and self-renewal potential suggests that HIF2A stabilization improves the long-term outcome of SC transplantation.

### Genetic ablation of HIF2A transiently improves muscle regeneration but impairs long-term muscle regeneration potential.

**Figure 6:**
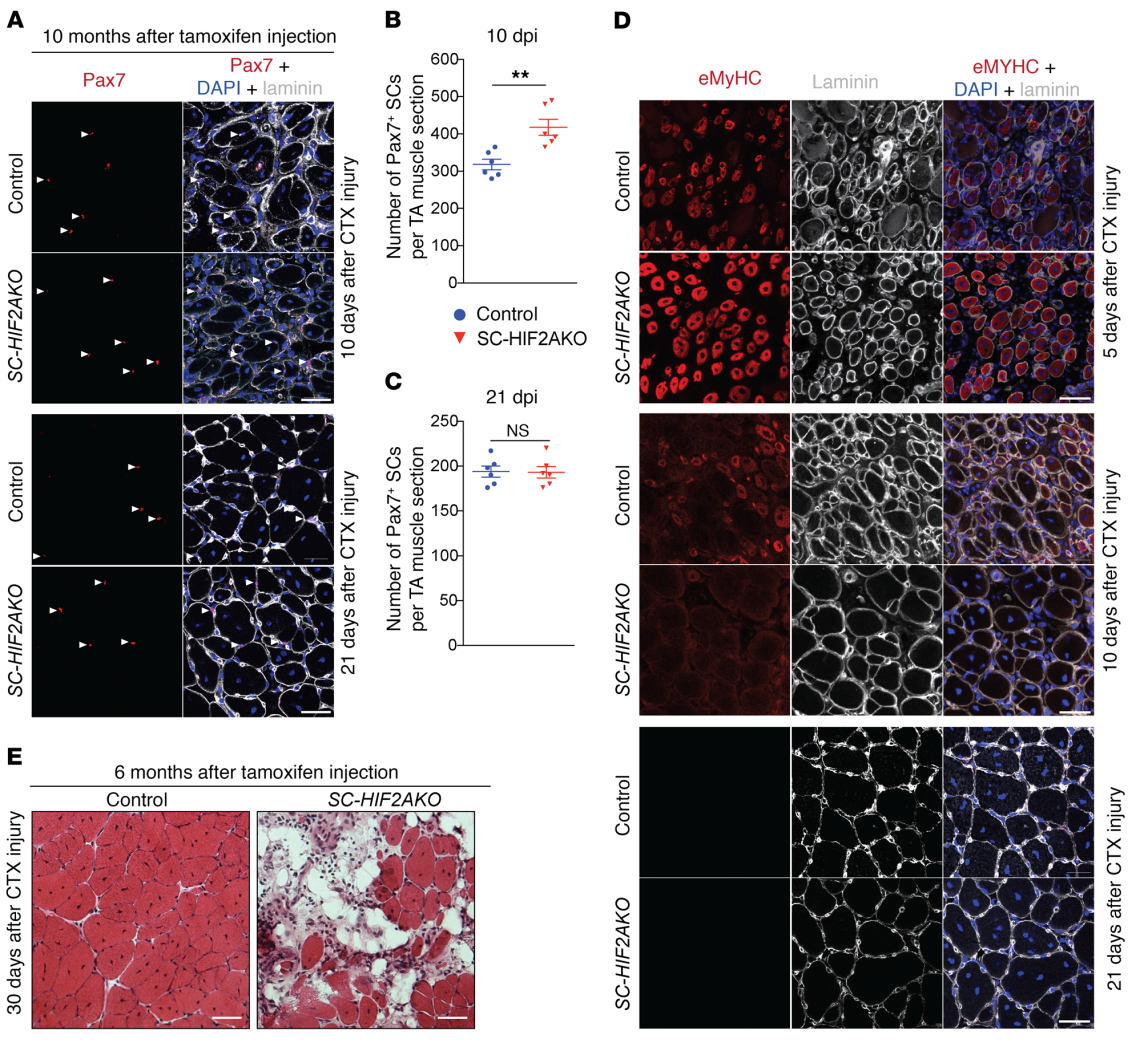
Genetic ablation of HIF2A transiently improves muscle regeneration but impairs long-term muscle regeneration potential. (**A**) Representative images of TA muscles from *SC-HIF2AKO* mice and control littermates (*n* = 6 mice/group/time point). The muscles were CTX injured 10 days after tamoxifen-induced HIF2A ablation (10 dpr). Immunofluorescence of Pax7 after CTX injury (10 and 21 dpi) revealed an increase in Pax7^+^ SCs (arrowheads) in *SC-HIF2AKO* mice. Scale bars: 20 μm. (**B** and **C**) Number of Pax7^+^ SCs per TA muscle section at 10 dpi (**B**) and 21 dpi (**C**). (**D**) Representative images of TA muscles from *SC-HIF2AKO* mice and control littermates (*n* = 6 mice/group/time point). The muscles were CTX injured 10 days after tamoxifen-induced HIF2A ablation (10 dpr). Immunofluorescence of eMyHC and laminin B2 days after CTX injury (5, 10, and 21 dpi) revealed accelerated muscle regeneration in *SC-HIF2AKO* mice. Scale bars: 20 μm. (**E**) Representative images of TA muscles from *SC-HIF2AKO* mice and control littermates (*n* = 3 mice/group). The muscles were CTX injured 6 months after tamoxifen-induced HIF2A ablation. H&E staining of TA muscles 30 days after CTX injury revealed impaired muscle regeneration in *SC-HIF2AKO* mice. Scale bars: 20 μm. **P < 0.01, by 2-sided Student’s *t* test. Data represent the mean ± SEM.

As HIF2A ablation in QSCs led to transient SC proliferation and myogenic differentiation, we sought to understand whether these effects may improve muscle repair. Stretch-induced injury is of physiological relevance, yet the diminished HIF2A expression in SCs after injury negates the necessity for genetic HIF2A ablation in this injury model. Unlike stretch-induced injury, HIF2A expression dampened but was still present in many SCs after CTX-induced injury (3 dpi; 3-month-old *C57BL/6* mice) (Supplemental Figure 5A); meanwhile, most SCs (along with many Pax7^–^ cells) expressed HIF1A, which is in line with reported massive blood vessel damage in CTX-injured muscles (Supplemental Figure 5A) (32). Thus, we examined TA muscle regeneration following CTX-induced injury in *SC-HIF2AKO* mice and their control littermates, which were administered tamoxifen 8 to 10 days before CTX injury (to deplete HIF2A in SCs from *SC-HIF2AKO* mice). SC-specific HIF2A ablation increased the number of Pax7^+^ SCs at 10 dpi in *SC-HIF2AKO* mice compared with that detected in control mice (control, ~320 SCs vs. *SC-HIF2AKO*; ~420 SCs per TA section) (Figure 6, A and B), indicating that HIF2A ablation transiently improved SC proliferation in regenerative muscle. Embryonic myosin heavy chain (eMyHC) was expressed at a higher level in newly formed/regenerating myofibers with centralized myonuclei in *SC-HIF2AKO* mice at 5 dpi, suggesting that HIF2A ablation also augments SC differentiation (Figure 6D). By 10 dpi, eMyHC had already diminished in most myofibers in *SC-HIF2AKO* mice yet was still present in many myofibers of small-caliber myofibers in the control mice, suggesting that regenerative myogenesis is accelerated in *SC-HIF2AKO* mice (Figure 6D). By 21 dpi, TA muscles from both *SC-HIF2AKO* and control mice contained fully regenerated myofibers (Figure 6D). The number of Pax7^+^ SCs in *SC-HIF2AKO* mice was comparable to that in control mice at 21 dpi, suggesting that the decrease in SC self-renewal may be compensated by the increase in proliferation in regenerative muscles (Figure 6, A and C). By 30 dpi, the percentages of myofibers expressing MyHC isoforms I, IIA, and IIB were comparable between *SC-HIF2AKO* and control mice (Supplemental Figure 5, B and C), indicating that SC-specific HIF2A ablation does not alter myofiber type composition during muscle regeneration.

Although HIF2A ablation in SCs transiently increases proliferation and myogenic differentiation and improves muscle regeneration, depletion of the SC pool after long-term HIF2A ablation expectedly impairs muscle regeneration instead. To confirm this, we injured TA muscles of *SC-HIF2AKO* and control mice with CTX 6 months after tamoxifen-induced HIF2A ablation, when SCs were depleted in *SC-HIF2AKO* mice (Figure 4B). At 30 dpi, the injured TA muscles in control mice were completely regenerated, whereas only a small number of regenerated myofibers were observed in *SC-HIF2AKO* mice (Figure 6E). The distinct outcomes of muscle regeneration in *SC-HIF2AKO* mice echo the transient and long-term effects of HIF2A ablation in SCs, namely that HIF2A ablation leads to transient increases in SC proliferation and myogenic differentiation, whereas HIF2A ablation in the long term results in loss of the SC population and regeneration failure.

### Transient pharmacological inhibition of HIF2A in CTX-injured muscle promotes SC proliferation and accelerates muscle regeneration.

**Figure 7:**
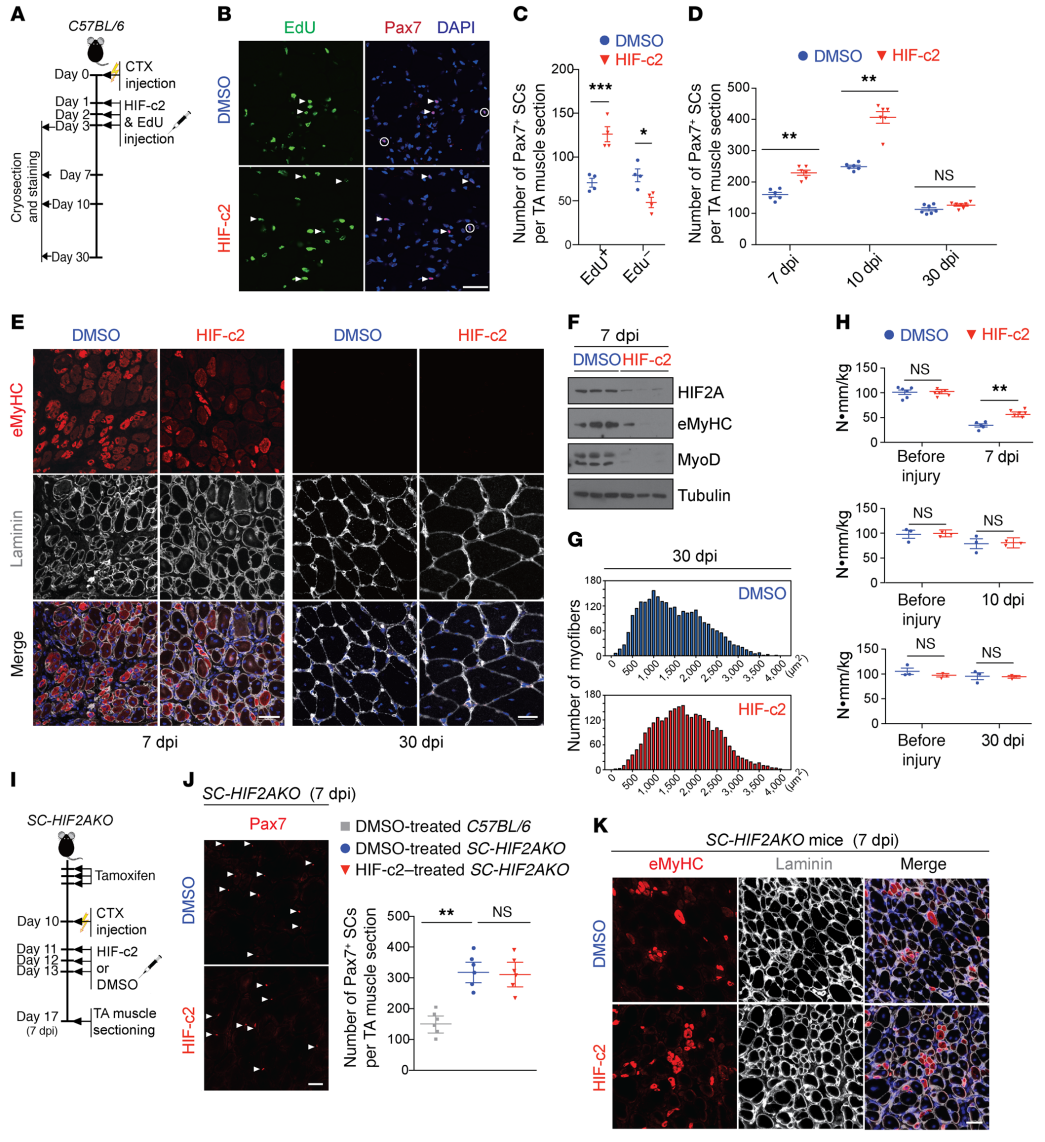
Transient pharmacological inhibition of HIF2A in CTX-injured muscle promotes SC proliferation and accelerates muscle regeneration. (**A**) Timeline of pharmacological inhibition of HIF2A after CTX-induced muscle injury in *C57BL/6* mice. (**B**) Representative images of HIF-c2– or 1% DMSO–treated TA muscles (3 dpi) from *C57BL/6* mice (*n* = 6 mice/group). Immunofluorescence revealed increased Pax7^+^EdU^+^ proliferative SCs (arrowheads) and decreased Pax7^+^EdU^–^ QSCs (circles). Scale bar: 20 μm. (**C**) Number of EdU^+^ and EdU^–^ SCs per TA muscle section 3 dpi (*n* = 6). (**D**) Number of Pax7^+^ SCs per TA muscle section 7 dpi, 10 dpi, and 30 dpi (*n* = 6 or 7). (**E**) Representative eMyHC and laminin B2 immunofluorescence in HIF-c2– or DMSO-treated TA muscles 7 dpi and 30 dpi (*n* = 3). Scale bars: 20 μm. (**F**) Immunoblots showing the expression levels of HIF2A, eMyHC, MyoD, and tubulin in HIF-c2– or DMSO-treated TA muscles 7 dpi (*n* = 3). (**G**) Distributions of myofiber cross-sectional areas of HIF-c2– or DMSO-treated TA muscles 30 dpi (*n* = 3). (**H**) Maximal torques of uninjured TA muscles and HIF-c2– or DMSO-treated TA muscles 7 dpi (*n* = 6), 10 dpi (*n* = 3), and 30 dpi (*n* = 3). (**I**) Timeline of pharmacological inhibition of HIF2A after CTX-induced muscle injury in *C57BL/6* mice and *SC-HIF2AKO* mice. (**J**)Representative Pax7 immunofluorescence images of HIF-c2– or DMSO-treated TA muscles from *SC-HIF2AKO* mice (*n* = 6 mice/group) 10 dpi and number of Pax7^+^ SCs per TA muscle section 7 dpi. Scale bar: 20 um. (**K**) Representative eMyHC and laminin B2 immunofluorescence images of HIF-c2– or DMSO-treated TA muscles from *SC-HIF2AKO* (*n* = 6 mice/group) 7 dpi. Scale bar: 20 μm. *P < 0.05, **P < 0.01, and ***P < 0.005, by 2-sided Student’s *t* test. Data represent the mean ± SEM.

The above observations suggest that a transient HIF2A inhibition following hypoxia-burdened muscle injury may benefit regeneration by promoting SC proliferation yet lessening the side effect of SC loss. To transiently inhibit HIF2A in muscle, we intramuscularly administered a HIF2A inhibitor (HIF-c2, CAS no. 1422955-31-4; 5 mg/injection/day over 3 consecutive days), which reduced HIF2A expression in muscle (including SCs) (Supplemental Figure 6A) as previously reported (33). We injected HIF-c2 or the carrier solution (1% DMSO) into CTX-injured TA muscles of adult *C57BL/6* mice (daily injections, 1–3 dpi) (Figure 7A). HIF-c2 treatment increased the number of Pax7^+^EdU^+^ proliferative SCs by 3 dpi and the number of Pax7^+^ SCs by 7 dpi and 10 dpi (Figure 7, B–D). By 7 dpi, HIF-c2–treated muscles contained regenerated myofibers of noticeably increased calibers and decreased expression of eMyHC (Figure 7E and Supplemental Figure 6B). Consistently, HIF-c2–treated muscles had reduced expression levels of HIF2A, the proliferative SC marker MyoD, and the early regeneration marker eMyHC (Figure 7F). Additionally, HIF-c2–treated muscles recovered approximately 53% of the maximal torque measured before injury, whereas the control muscles only recovered approximately 32% of the maximal torque by 7 dpi (Figure 7H), indicating accelerated muscle regeneration. At 30 dpi, when muscle regeneration was completed, both HIF-c2–treated and control muscles contained comparable numbers of Pax7^+^ SCs and recovered approximately 90% of their maximal torque (Figure 7, D and H), suggesting that the HIF-c2 treatment regimen does not affect SC self-renewal or the future contractile capacity of regenerated muscle. However, regenerated myofibers in HIF-c2–treated muscles had increased myofiber calibers (Figure 7, E and G). HIF-c2–treated muscles had no obvious sign of fibrosis and had a myofiber type composition similar to that of control muscles (Supplemental Figure 6, C–E). Therefore, transient inhibition of HIF2A after CTX-induced muscle injury accelerates the progression of muscle regeneration.

As HIF-c2 treatment is not specific to SCs, we asked whether the beneficial effects are attributable to HIF2A inhibition in SCs. Thus, we repeated the same HIF-c2 treatment regimen (with 1% DMSO as a control) in CTX-injured TA muscles from *SC-HIF2AKO* mice, in which HIF2A had been genetically ablated in SCs (CTX injury at 10 dpr) (Figure 7I). By 7 dpi, DMSO-treated muscles from *SC-HIF2AKO* mice had increased numbers of Pax7^+^ SCs compared with DMSO-treated muscles from WT mice (Figure 7J). However, in *SC-HIF2AKO* mice, HIF-c2 treatment did not further increase the number of Pax7^+^ SCs by 7 dpi (compared with DMSO-treated *SC-HIF2AKO* mice) (Figure 7J). In addition, eMyHC levels and the morphology of regenerating myofibers were comparable in HIF-c2– and DMSO-treated muscles of *SC-HIF2AKO* mice (Figure 7K). Thus, the beneficial effects of HIF-c2 on muscle regeneration in WT mice were probably attributable to HIF2A inhibition in SCs.

### Spry1 is a target of HIF2A in QSCs.

**Figure 8:**
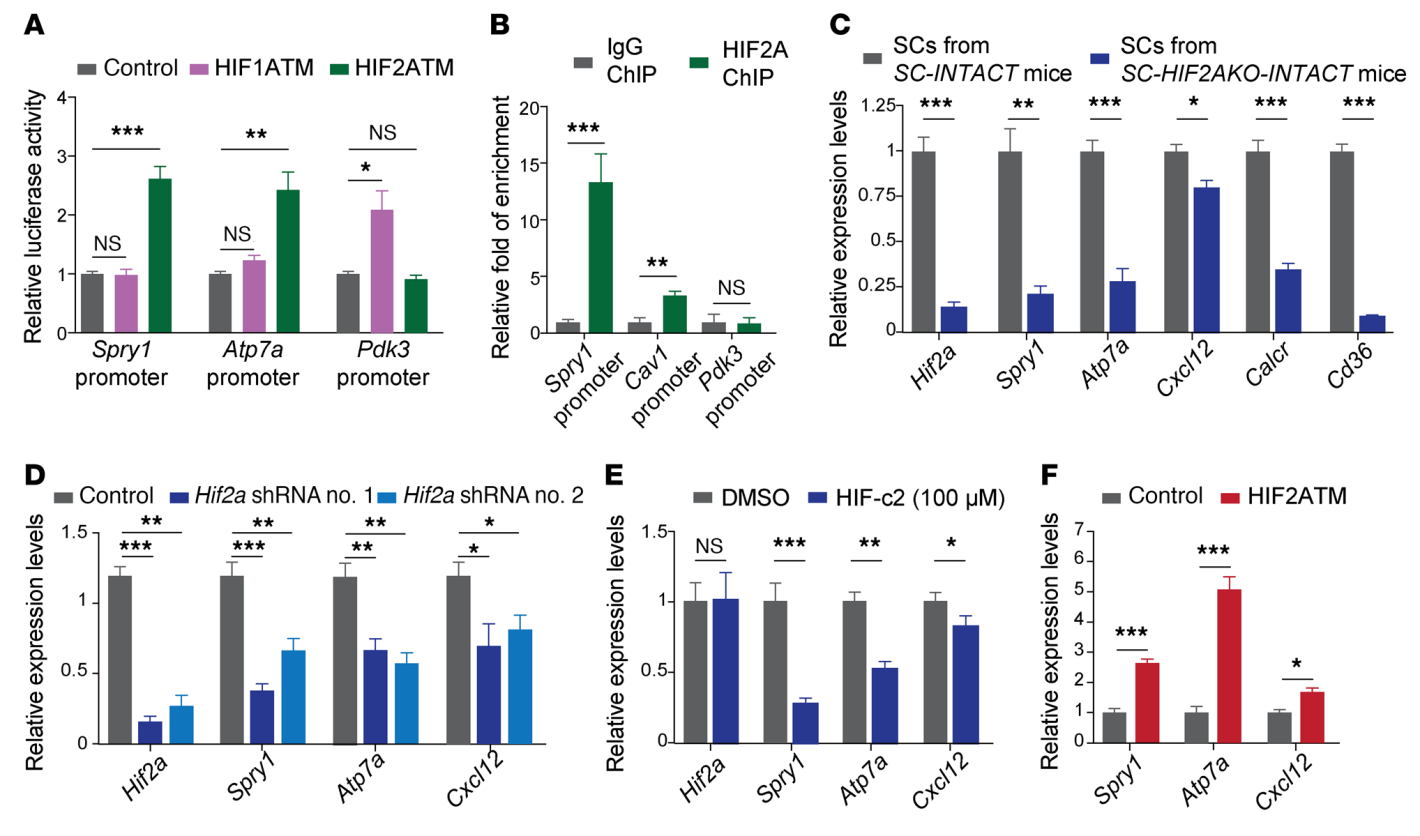
*Spry1* is a target of HIF2A in QSCs. (**A**) Luciferase assays showed that stabilized expression of HIF2A (HIF2ATM), but not HIF1A (HIF1ATM), increased the promoter activities of *Spry1* and *Atp7a* (a known HIF2A target). In contrast, HIF1A, but not HIF2A, transactivated the *Pdk3* promoter. (**B**) ChIP-qPCR indicated that HIF2A bound the promoters of *Spry1* and *Cav1* (a known HIF2A target), but not the *Pdk3* promoter, in QSCs in vivo. (**C**) RT-qPCR revealed that HIF2A ablation in QSCs in vivo reduced the mRNA levels of *Hif2a*, *Spry1*, *Calcr*, and *Cd36*. (**D**) RT-qPCR indicated that 2 HIF2A shRNAs reduced the mRNA levels of *Hif2a* and *Spry1* as well as of 2 known HIF2A targets, *Atp7a* and *Cxcl12*, in C2C12 myoblasts. (**E**) RT-qPCR revealed that HIF-c2 treatment decreased mRNA levels of the HIF2A targets *Spry1*, *Atp7a*, and *Cxcl12* in C2C12 myoblasts. (**F**) RT-qPCR indicated that HIF2ATM increased the mRNA levels of *Spry1* as well as of 2 known HIF2A targets, *Atp7a* and *Cxcl12*, in C2C12 myoblasts. *P < 0.05, **P < 0.01, and ***P < 0.005, by 2-sided Student’s *t* test. Data represent the mean ± SEM.

Spry1 is highly expressed in QSCs, but not in proliferative SCs, and is essential for the self-renewal and maintenance of SCs (13, 34). Bioinformatic analysis identified 3 conserved HREs within the proximal promoter of the *Spry1* gene (Supplemental Figure 7A), suggesting that HIF2A promotes SC self-renewal by activating Spry1. In luciferase assays, the HRE-containing *Spry1* promoter was specifically responsive to stabilized HIF2A, but not stabilized HIF1A, in C2C12 myoblasts (Figure 8A). *Atp7a* and *Pdk3* are specific target genes of HIF2A and HIF1A, respectively (15, 35, 36). Consistently, an *Atp7a* promoter was specifically responsive to stabilized HIF2A, whereas a *Pdk3* promoter was only responsive to stabilized HIF1A.

To confirm that HIF2A at its native expression level directly binds to the *Spry1* promoter under hypoxia, we performed HIF2A ChIP using 2 × 10^6^ nmGFP^+^ QSC nuclei that were fixed in vivo and isolated from *SC-INTACT* mice (Supplemental Figure 7B and see Methods). In support of the efficacy and specificity of HIF2A ChIP in QSCs, ChIP-qPCR revealed that a *Cav1* promoter, which was previously shown to be associated with HIF2A in hypoxic colorectal epithelial cells (37), was also enriched by 3.4 fold in QSC HIF2A ChIP samples, whereas no enrichment was detected for the *Pdk3* promoter (Figure 8B). ChIP-qPCR also revealed an approximately 13-fold enrichment of the *Spry1* promoter in HIF2A ChIP samples, indicating that HIF2A directly binds to the *Spry1* promoter in QSCs (Figure 8B).

To investigate whether HIF2A activates *Spry1* in QSCs, we isolated nmGFP^+^ SCs from *SC-INTACT* and *SC-INTACT-HIF2AKO* mice (at 10 dpr) by FACS and performed reverse transcription quantitative PCR (RT-qPCR). SCs from *SC-INTACT-HIF2AKO* mice had reduced expression of *Hif2a* and the QSC markers *Calcr* and *Cd36* (Figure 8C) (8, 38), confirming that HIF2A ablation leads to SC activation. Importantly, HIF2A-ablated SCs expressed decreased levels of *Spry1* and the HIF2A targets *Atp7a* and *Cxcl12* (Figure 8C) (39), indicating that HIF2A transactivates *Spry1* (and *Atp7a* and *Cxcl12*) in SCs.

We further investigated whether *Spry1* expression is responsive to HIF2A expression levels in vitro. Under normoxia, 2 HIF2A-targeting shRNAs independently knocked down *Hif2a* (but not *Hif1a*) in C2C12 myoblasts, which also decreased the expression of *Spry1*, *Atp7a,* and *Cxcl12* (Figure 8D and Supplemental Figure 7C). Likewise, HIF-c2, although not affecting *Hif2a* mRNA levels, reduced HIF2A protein levels in C2C12 myoblasts as well as decreased the mRNA levels of *Spry1*, *Atp7a,* and *Cxcl12* (Figure 8E and Supplemental Figure 7D). Instead, HIF-c2 increased the levels of *Hif1a* mRNA and HIF1A protein and mRNA levels of the known HIF1A targets *Pdk3* and *Slc2a1* (also known as *Glut1*) (Supplemental Figure 7, D and E) (40). Conversely, overexpression of HIF2ATM in C2C12 myoblasts increased the mRNA levels of *Spry1*, *Atp7a,* and *Cxcl12* (Figure 8F and Supplemental Figure 7F). Thus, *Spry1* is an HIF2A target gene, and HIF2A transactivates *Spry1* in SCs and myoblasts.

## Discussion

This study uncovers a previously unappreciated hypoxic state of SCs in their native niche. Pimonidazole and CCI-103F form nonspecific protein adducts when pO_2_ is less than 1.3% (or 10 mmHg) and have been widely used to determine hypoxic states of stem cells and tumor cells in vivo (41–43). QSCs in healthy resting muscle stained positive for pimonidazole and CCI-103F, indicating that SCs are intracellularly hypoxic. This finding echoes a previous observation that SCs isolated from postmortem muscle retain their viability and regenerative potential (44) and implies that SCs adapt to hypoxia. The pO_2_ in healthy resting muscle ranges from 3% to 4% (25–34 mmHg) (24, 45, 46). Consistently, we found that the sarcoplasm of myofibers had much less evident staining for pimonidazole and CCI-103F. The distinct pO_2_ in SCs and myofibers is intriguing, as these 2 types of cells are juxtaposed. It has been reported that SCs are preferentially localized adjacent to capillaries (47). Thus, the hypoxic state of SCs is probably not the result of poor oxygenation in their extracellular milieu, but rather is due to an unknown intrinsic mechanism. Notably, hematopoietic stem cells in the bone marrow are also pimonidazole^+^, irrespective of the variable distance from the vasculature or local pO_2_ (1%–4%) and are distinct from adjacent pimonidazole^–^ cells (48, 49). Thus, a hypoxic state seems to be physiologically inherent to some adult stem cell populations, which is suggestive of an obligatory role of hypoxia in stem cell biology.

Mammalian cells sense and cope with hypoxia by stabilizing HIFs (HIF1A, HIF2A, and HIF3A). In line with the hypoxic state, QSCs express HIF2A, but not HIF1A. This finding is novel but not surprising (50). Recent studies demonstrated that HIF1A and HIF2A, although sharing many common targets, often have disparate or even opposite functions (50). In particular, HIF1A promotes glucose uptake, augments glycolysis flux, and reduces glucose oxidation in the TCA cycle under hypoxia or pseudohypoxia, all of which favor biosynthesis and cell proliferation in many types of fast-cycling cells, such as hematopoietic stem cells and many types of cancer cells (50–52). In contrast, HIF2A promotes stem cell characteristics in multiple types of cancer stem cells, and HIF2A reexpression suppresses the growth of soft tissue sarcoma (53–55). In addition, HIF2A enhances the stemness of human embryonic stem cells and the reprogramming efficiency of human induced pluripotent stem cells (15, 41). Thus, it is conceivable that the selective stabilization of HIF2A (but not HIF1A) in SCs ensures their quiescence and stemness. Indeed, HIF2A-ablated SCs proceed to proliferate, differentiate, and eventually become depleted in uninjured muscles, reflecting a pivotal role of HIF2A in the maintenance of this adult stem cell population.

This study also revealed intriguing expression patterns of HIF2A and HIF1A in SCs during muscle repair: after stretch muscle injury, HIF2A was diminished in SCs, followed by SC proliferation and transient HIF1A expression in a subset of SCs; in contrast, after CTX-induced injury, most SCs expressed both HIF2A and HIF1A. The emerging HIF1A expression in SCs apparently correlates with vascular damage in muscle and local hypoxia and ischemia. In support of this view, it has been shown that HIF2A controls chronic hypoxia signaling under medium-level hypoxia, whereas HIF1A mediates acute hypoxia responses under transient extreme hypoxia (53, 54). However, it is notable that the above-mentioned HIF1A/HIF2A dynamics cannot be the sole result of HIF-α stabilization/destabilization in response to tissue oxygenation, as evidenced by the findings that (a) following HIF2A depletion, HIF1A emerged in a small percentage of SCs in uninjured, resting muscles, wherein local pO_2_ probably remained unchanged; and (b) after stretch-induced muscle injury, most SCs remained pimonidazole^+^, yet HIF2A expression had diminished. Instead, these observations strongly implicate O_2_-independent mechanisms in HIF1A and HIF2A regulation in SCs. Notably, the global translation profile of QSCs markedly differs from that of activated SCs (56), and the translation of *Hif2a* mRNA (but not *Hif1a* mRNA) is regulated by the insulin receptor/PI3K/mTORC2 pathway and IREBP1 (57, 58). Thus, it is conceivable that HIF1A and HIF2A expression in SCs may be regulated at the translational level during muscle repair, an idea that warrants further investigation in the future.

This study shows consistent evidence of SC-specific gain and loss of function of HIF2A and supports the notion that HIF2A promotes SC quiescence or a return to quiescence (self-renewal), while it impedes the myogenic differentiation of these cells. Given that self-renewal, proliferation, and differentiation divergently affect SC numbers, we found that HIF2A ablation led to distinct consequences in terms of the size of the SC pool and muscle regeneration capacity. Shortly after HIF2A ablation, SCs transiently expanded in number and proceeded to myogenic differentiation, which benefits regeneration, with increased input from the stem cell compartment. In contrast, chronic HIF2A loss in SCs skewed the self-renewal versus differentiation propensities and exhausted the stem cell pool, which was destructive to muscle regeneration.

Reduced stemness and the consequent poor long-term engraftment potential of in vitro–expanded myoblasts presents one of the major hurdles for SC-based therapies for degenerative muscle diseases (59). Myoblasts cultured under hypoxia have increased quiescence, self-renewal, and engraftment efficiency (17). In this study, HIF2A stabilization in normoxic culture elicited the same series of effects as hypoxia. Intriguingly, HIF2A-stabilized SCs outperformed the control transplanted SCs and the endogenous SCs in terms of their self-renewal capability and overall contribution to the myonuclear compartment after repetitive regeneration. It seems paradoxical that HIF2A promotes SC quiescence and also increases the number of engrafted SCs. It is also apparently conflicting that HIF2A impeded myogenic differentiation, yet HIF2A-stabilized SCs gave rise to more myonuclei. However, it is notable that a small percentage of SCs preferentially undergo asymmetric cell divisions, divide slowly, and withdraw early from the cell cycle (11, 60–62). When transplanted, these SCs have long-term self-renewal ability and can efficiently regenerate injured muscles, which are characteristics of true stem cells (60). In this perspective, the above apparent conflicting observations may actually reflect the increased stemness of HIF2A-stabilized SCs, characterized by the improved ability to maintain a quiescent and undifferentiated stem cell state as well as to sustain a myogenic progenitor pool and support muscle regeneration in the long term. Thus, compared with in vitro hypoxia treatment, HIF2A stabilization could afford lasting stemness enhancement and long-term engraftment of myoblasts in transplantation-based therapies for degenerative muscle diseases.

This study also revealed that Spry1 is a target of HIF2A in SCs. Like *Hif2a*, *Spry1* mRNA is abundant in QSCs and downregulated in proliferative SCs, and Spry1 ablation in SCs results in a loss of SC quiescence in uninjured muscle and impaired SC self-renewal during muscle regeneration (13, 34). These similar expression patterns and phenotypes suggest a genetic interaction between Spry1 and HIF2A. In this study revealed strong evidence that HIF2A binds to the *Spry1* promoter and transactivates *Spry1*. Interestingly, HIF1A appeared not to transactivate *Spry1*, which is in line with its absence in QSCs. Previous studies showed that HIF1A and HIF2A require distinct coactivators for transcriptional activation (63, 64). Further investigation of specific targets and coactivators of HIF1A and HIF2A in SCs would be instrumental in elucidating their distinct functions in SCs.

Various types of muscle injury involve different levels of vascular damage and expectedly have distinct effects on HIF expression in SCs (32). We postulate that the vasculature in regenerative muscle coordinates with SCs via hypoxia signaling to meet distinct needs for SC proliferation upon different types of muscle injury. Understanding the hypoxia signaling in regenerative muscle may shed light on specific therapeutic strategies for muscle injuries complicated with different levels of hypoxia and/or ischemia. In this study, we demonstrate that HIF2A loss of function benefited muscle regeneration after CTX-induced injury, which involved vascular damage and remodeling and heterogeneous HIF2A reduction in activated SCs. HIF2A inhibition augmented SC proliferation and accelerated myogenic differentiation, apparently alleviating a negative impact of hypoxia/ischemia on muscle regeneration capacity. The beneficial effects of HIF2A inhibition are probably not the result of a compensatory expression of HIF1A, given that SC-specific HIF1A ablation has been shown to increase myogenic differentiation without an effect on SC expansion following ischemic muscle injury (18). Additionally, SC-specific ablation of both HIF1A and HIF2A impairs muscle regeneration, with reduced SC expansion and self-renewal (19). A previous study reported that *myogenin Cre*–mediated HIF2A ablation results in myofiber switching from slow to fast types (65). In addition, muscle regeneration with HIF1A ablation in SCs ends in muscle fibrosis (18). In this study, we did not observe these side effects following transient HIF2A inhibition in regenerative muscle. Given the accelerated recovery of muscle contractile function, transient HIF2A inhibition represents an appealing strategy for improving muscle regeneration after hypoxia/ischemia-involved muscle injury.

## Methods

### Animal experimental procedures.

All animals were housed at 23°C under 12-hour light/12-hour dark cycles with ad libitum access to food and water. All mouse strains were purchased from The Jackson Laboratory (*Pax7^CreERT2^*, no. 017763; *R26R^CAG-Sun1/sfGFP^,* no. 021039; and *Hif2a^fl^* , no. 008407).

To induce Cre activity, tamoxifen (20 mg/ml in corn oil) was administered i.p. for 3 consecutive days (100 mg/kg/d). To assess myofiber damage, 1% Evans blue (4 ml/kg in saline) was administered i.p. To trace proliferation, EdU (50 mg/kg in DMSO) was administered i.p. For induction of muscle injury, CTX (0.5 nmol, 100 μl) was injected into TA muscles.

### Plasmid construction.

For HIF1ATM/HIF2ATM expression, pcDNA3_mHIF2A_MYC and pcDNA3_mHIF1A_MYC plasmids were purchased from Addgene (nos. 44027 and 44028). HIF ORFs were subcloned into pCDH-CMV-MCS-EF1-eGFP-IRES-Puro plasmid (pCMEGIP; System Biosciences). For HIF2AWT expression, the mHIF2A ORF was PCR amplified from a mouse cDNA library using the primers HIF2A_ORF_S (5′-ACAGCTGACAAGGAGAAAAAAAGGAG-3) and HIF2A_ORF_AS (5′-GGTGGCCTGGTCCAGAGC-3′) and cloned into the pCMEGIP plasmid.

For transplantation of HIF2ATM-transfected SCs and myofibers, the mHIF2ATM ORF was subcloned into the pCDH-CMV-MCS-EF1-Puro plasmid (pCMEP; System Biosciences), which does not express GFP.

For HIF2A knockdown, HIF2A shRNA oligonucleotide no. 1 (5′-TGCTGTTGACAGTGAGCGAACACTTGATGTGGAAACGTATTAGTGAAGCCACAGATGTAATACGTTTCCACATCAAGTGTGTGCCTACTGCCTCGGA-3′) and HIF2A shRNA oligonucleotide no. 2 (5′-TGCTGTTGACAGTGAGCGATCCAACAAGCTGAAGCTAAAGTAGTGAAGCCACAGATGTACTTTAGCTTCAGCTTGTTGGACTGCCTACTGCCTCGGA-3′) were cloned into pMSCV-P2GM plasmids (Addgene; no. 19750).

### In situ detection of hypoxic SCs by hypoxyprobes.

Hypoxyprobe-1 (pimonidazole; Hypoxyprobe Inc.; 60 mg/kg in PBS) and Hypoxyprobe-F6 (CCI-103F; Hypoxyprobe Inc.; 60 mg/kg in DMSO) were administered i.p. 1.5 and 3 hours before euthanasia, respectively. To assess Hypoxyprobe-1 in staining hypoxic myonuclei, myofibers from tamoxifen-induced *Pax7^Cre/ERT2^* *R26R^CAG-Sun1/sfGFP^* mice were cultured in a hypoxia chamber (STEMCELL Technologies) supplied with 1% O_2_ and 5% CO_2_ for 20 hours and in the presence of Hypoxyprobe-1 (100 μM) for 2 hours.

### Maximal contraction force measurement and eccentric contraction–induced injury.

The peak isometric torque of the ankle dorsiflexors was assessed as previously described (66). Briefly, the left foot of anesthetized mice was placed on a foot plate attached to a servomotor (model 300C-LR; Aurora Scientific). Two Pt-Ir electrode needles (model E2-12; Grass Technologies) were inserted percutaneously into either side of the peroneal nerve. The ankle joint was secured at a 90° angle. The peak isometric torque was achieved by varying the current delivered to the peroneal nerve at a frequency of 200 Hz and a 0.1-ms square wave pulse. Torque (N•mm) was normalized by the body mass (kg) to account for differences in body size.

To induce injury, the dorsiflexors were subject to 100 electronically stimulated eccentric contractions, during which the foot was passively moved from the 0° position (perpendicular to the tibia) to 20° of dorsiflexion. The dorsiflexors were stimulated at 200 Hz for a 100-ms isometric contraction followed by an additional 50-ms stimulation while moving from 20° dorsiflexion to 20° plantarflexion at an angular velocity of 800°/s. Eccentric contractions were repeated every 10 seconds.

### Myofiber isolation, culture, and transfection.

EDL myofibers were isolated as described before (67). Briefly, EDL was dissected and digested in 0.2% Type I Collagenase (Worthington) in DMEM at 37°C for 1.5 hours. Single myofibers were isolated by triturating the digested EDL muscle with polished Pasteur pipettes. Myofibers were cultured in horse serum–coated 24-well plates in DMEM (4.5 g/l glucose) supplemented with 20% FBS, 1% sodium pyruvate, and 1% chicken embryo extract and 1% penicillin-streptomycin. For myofiber transfection, myofibers were cultured for 12 hours and transfected with 2 μg plasmids with Lipofectamine 3000 (Thermo Fisher Scientific) for 6 hours.

### SC and myofiber transplantation.

Myofibers were isolated from tamoxifen-induced *SC-INTACT* mice, cultured for 12 hours, transfected with plasmids for 6 hours, and briefly washed. Ten transfected myofibers were loaded into a twenty-eight–gauge insulin syringe and injected into each TA muscle that was damaged by CTX twenty-four hours beforehand.

### Myoblast culture, transfection, and differentiation.

Primary myoblasts were isolated from 1-week-old *C57BL/6* male mice as described previously (68). Primary myoblasts were cultured on collagen-coated dishes in Ham’s F-10 medium supplemented with 20% FBS, 5 μg/l basic FGF, and 1% penicillin-streptomycin. C2C12 myoblasts were purchased from ATCC and cultured in DMEM (4.5 g/l glucose) medium supplemented with 10% FBS and 1% penicillin-streptomycin. For hypoxia treatment, primary myoblasts and C2C12 myoblasts were cultured in a hypoxia chamber supplied with 1% O_2_ and 5% CO_2_. C2C12 myoblasts were transfected with plasmids using TransIT-X2 (Mirus Bio). C2C12 myoblasts (100% confluent) were differentiated in DMEM (4.5 g/l glucose) medium supplemented with 2% horse serum for 5 days.

### Cell-cycle analysis of primary myoblasts.

Primary myoblasts were infected with lentivirus packaged from a pCMEGIP-HIF2aTM plasmid or a control pCMEGIP empty plasmid, selected with puromycin (0.5 μg/ml) for 1 week, fixed with 70% ethanol for 10 minutes, and stained with Hoechst 33342 (10 μg/ml). Myoblasts were run into a HyperCyAn Cytometer (Beckman Coulter), and profiles were analyzed with FlowJo, version 10.

### FACS.

SC sorting was performed on a MoFlo XDP Cell Sorter (Beckman Coulter). nmGFP^+^ SCs (~2 × 10^5^) from 3-month-old *SC-INTACT* and *SC-HIF2AKO-INTACT* mice (7 dpr) were FACS sorted as previously described (68).

### Immunofluorescence staining and imaging.

For staining of muscle sections, muscle sections were fixed with paraformaldehyde (PFA)/PBS (1%, 10 min), quenched with glycine (50 mM, 10 min), permeabilized with Triton X-100 (0.5%, 10 min), blocked with Mouse On Mouse (M.O.M.) Blocking Reagent (Vector Laboratories) and 5% BSA/5% normal goat serum/PBS, and incubated with the following primary antibodies: anti-Pax7 (1:5; Developmental Studies Hybridoma Bank [DSHB] Pax7); anti-HIF2A (1:250; Novus Biologicals; NB100-122); anti-HIF1A (1:250; Novus Biologicals; NB100-105); anti-Ki67 (1:1,000; Abcam; ab15580); anti–laminin B2 (1:1,000; MilliporeSigma; 05-206); anti-eMyHC (1:50; DSHB; F1.652); and anti–MyHC type I, IIA, or IIB (1:50; DSHB; BA-D5; SC-71; BF-F3) overnight at 4°C. For EdU staining, a Click-iT EdU Fluorescence Kit (Life Technologies, Thermo Fisher Scientific) was used according to the manufacturer’s instructions before primary antibody application. Sections were washed in PBS/0.1% Tween-20, incubated with Alexa Fluor–labeled secondary antibodies (1:200, 1 h), and then washed and mounted with DAPI-containing mounting medium (Life Technologies, Thermo Fisher Scientific).

For staining of myofibers and myoblasts, the myofibers and myoblasts were fixed in PFA (4%, 10 min), blocked in 5% BSA/5% normal goat serum/PBS, and incubated with the following primary antibodies: anti-Pax7 (1:50); anti-MyoD (1:250; MilliporeSigma; M6190); anti-HIF2A (1:250); anti-HIF1A (1:250); and anti-GFP (1:1,000; 101Bio; P601). Mounted slides were imaged on a Zeiss LSM 710 confocal microscope.

### TUNEL labeling.

Muscle sections were stained using a FlowTACS Apoptosis Detection Kit (Thermo Fisher Scientific) following the manufacturer’s instructions. Briefly, sections were fixed by formaldehyde (3.7%, 10 min), washed with PBS, incubated with 20 μl Cytonin solution (Thermo Fisher Scientific) for 30 minutes, and washed with PBS. At this step, sections assigned for positive controls were further incubated with 25 μl TACS-Nuclease (Thermo Fisher Scientific) solution at room temperature for 30 minutes. All sections were washed with 1× labeling buffer, incubated with the labeling reaction mix (1 μl 50× MnCl_2_, 1 μl TdT dNTP mix, 1 μl TdT enzyme, and 50 μl 1× labeling buffer) at room temperature for 1 hour, incubated with 1× stop buffer, and then washed in PBS. All sections were incubated with DAPI and streptavidin–Alexa Fluor 647 (1:200) for 10 minutes in the dark and then washed and mounted for confocal microscopy.

### Picrosirius red staining.

Muscle sections were stained with Picrosirius red solution (0.1% in picric acid, 15 min), washed with acetic acid (1%, 3 times), and mounted with Cytoseal 60 Mounting Medium (Thermo Fisher Scientific).

### SC nuclei isolation.

SC nuclei were isolated by isolation of nuclei tagged in specific cell types (INTACT). *SC-INTACT* mice were administered tamoxifen 1 week before nuclei isolation to induce sfGFP- and Myc-tagged Sun1 localization on SC nuclear membranes. Anti-GFP (2.5 μg; 101Bio; no. P601) and anti-Myc (2.5 μg; MilliporeSigma; no. 05-419) antibodies were incubated with Protein A Dynabeads (50 μl 50% slurry; Thermo Fisher Scientific) in PBST on a rotating platform at 4°C overnight. To cross-link HIF2A in situ on SC chromatin in vivo, *SC-INTACT* mice were transcardially perfused with 30 ml 0.5% PFA and 30 ml of 125 mM glycine as previously described (69). Limb and trunk muscles were dissected, ground in a mortar with liquid nitrogen, and homogenized (15 ml Wheaton dounce, loose pestle) in homogenization buffer (250 mM sucrose, 2 mM MgCl_2_, 25 mM KCl, 1% NP40) for 30 strokes. The homogenate was filtered using 100-μm and 40-μm strainers and centrifuged at 1,000 *g* at 4°C for 5 minutes to collect crude nuclei. Nuclei were resuspended in 10 ml homogenization buffer and loaded on top of a 2-step buffer cushion (lower buffer: 500 mM sucrose, 2 mM MgCl_2_, 25 mM KCl and 40% glycerol; upper buffer: 340 mM sucrose, 2 mM MgCl_2_, 25 mM KCl, and 40% glycerol). The nuclei were centrifuged in a swing bucket rotor at 1,000 *g* at 4°C for 10 minutes. Nuclei enriched at the interface were collected with long-neck Pasteur pipettes and checked under a fluorescence microscope. The above homogenization and centrifugation procedure was repeated until most nmGFP^+^ SC nuclei were physically separated from tissue debris or other nmGFP^–^ nuclei. Nuclei were incubated with antibody-coated Dynabeads in INTACT IP buffer (340 mM sucrose, 2 mM MgCl_2_, 25 mM KCl, and 5% glycerol) on a rotating platform (8 rpm/min) at 4°C overnight. Nuclei tethered on Dynabeads were isolated on magnetic stands and washed (~6 times) with PBS to remove the nonbound nmGFP^–^ nuclei. After each round of washing, the yield and purity of nmGFP^+^ SC nuclei were assessed under a microscope.

### ChIP and ChIP-qPCR.

INTACT-purified SC nuclei (with Dynabeads) were resuspended in 100 μl lysis buffer (1% SDS, 10 mM EDTA, 50 mM Tris HCl, pH 8.1, 1× protease inhibitor cocktail) and sonicated at 4°C in a Bioruptor Pico Sonicator (Diagenode) using 15 on/off cycles of 30:30 seconds. The sheared chromatin (~300 bp) was 1:10 diluted in IP dilution buffer (50 mM HEPES-KOH, pH7.5, 140 mM NaCl, 1 mM EDTA, 1% Triton X-100, 0.1% sodium deoxycholate, 0.1% SDS) and centrifuged at 20,000 *g* at 4°C for 10 minutes. The supernatant was transferred to siliconized tubes and incubated with 2 μg HIF2A antibody (Novus Biologicals) or rabbit IgG (Santa Cruz Biotechnology) on a rotating platform at 4°C overnight. PBS-washed Protein A Dynabeads (30 μl 50% slurry) were added to the chromatin-antibody mixture and incubated on a rotating platform at 4°C for 4 hours. All ChIP beads were sequentially washed with low salt, high salt, lithium, and TE buffer 2 times each and reverse cross-linked with 1 M NaCl for 6 hours at 65°C. Samples were digested with 10 μg RNase A (37°C, 0.5 h) and 20 μg proteinase K (55°C, overnight). Genomic DNA (gDNA) for ChIP analysis was purified by phenol/chloroform/isoamyl alcohol (25:24:1) extraction followed by precipitation with 100% isopropanol, 0.3 M sodium acetate, and GlycolBlue (Thermo Fisher Scientific) at –20°C overnight. The following primers were used to quantify relative enrichment levels at HRE-flanking regions in promoters of *Spry1*, *Cav1*, and *Pdk3* genes in the mouse genome: Spry1_promoter_S (5′-GAGTGTCCTGGGTTCCTTGC-3′); Spry1_promoter_AS (5′-GGCATAATGCATTTGCAAGC-3′); Cav1_promoter_S (5′-CCTTGGGGATGTGCCTAG-3′); Cav1_promoter_AS (5′-CAGGGGTTTGTTCTGCTCT-3′); Pdk3_promoter_S (5′- CCGCGACACCTACACAAG-3′); and Pdk3_promoter_AS (5′- TTACCGGGGCTTTAAGGAAG-3′).

The following primers were used for PCR amplification of gene-lacking regions on chromosomes 5 and 6, which serve as inner reference controls in the calculation of relative enrichment levels: Chr5_S (5′-CCCGTCACTCAACCATTTCA-3′); Chr5_AS (5′-CTTATCAATGGGGGCTCTGG-3′); Chr6_S (5′-AGATATGGCTGGCTTTGTGC-3′); and Chr6_AS (5′-GAACTCGCTCAGGTTCTGC-3′).

### RT-qPCR.

Total RNA was isolated from C2C12 myoblasts using TRIzol or from FACS-sorted SCs using a PicoPure RNA Isolation Kit (both from Thermo Fisher Scientific). Total RNA (1 μg from C2C12 myoblasts or 0.2 μg from SCs) was used in 20 μl reverse transcription reactions (Maxima; Life Technologies, Thermo Fisher Scientific) and diluted at 1:5. RT-qPCR reactions were performed with SsoAdvanced Universal SYBR Green Mix (Bio-Rad) on a Bio-Rad CFX384 PCR System. Relative expression values (normalized to *Rps18* and *Tbp*) were calculated using BioRad CFX Manager Software. The following primers were used for RT-qPCR: HIF2A_S (5′-ACATGGCCCCCGATGAAT-3′); HIF2A_AS (5′-CAGGTAAGGCTCGAACGATG-3′); HIF1A_S (5′-ATAGCTTCGCAGAATGCTCAG-3′); HIF1A_AS (5′-CAGTCACCTGGTTGCTGCAA-3′); Spry1_S (5′-GGATTTGGCCGAGAGTTGTT-3′); Spry1_AS (5′-CGGCTAGGAGAAGGACACTA-3′); Atp7a_S (5′-AAAGAAGAGGTCGGACTGCT-3′); Atp7a_AS (5′-AATGCCAACCTGAGAAGCAAT-3′); Cxcl12_S (5′-TGCATCAGTGACGGTAAACC-3′); Cxcl12_AS (5′-TGGGCTGTTGTGCTTACTTG-3′); Slc2a1_S (5′-CAGTTCGGCTATAACACTGGTG-3′); Slc2a1_AS (5′-GCCCCCGACAGAGAAGATG-3′); Calcr_S (5′-CGGCGGGATCCTATAAGTTG-3′); Calcr_AS (5′-GCGTGGATAATGGTTGGCA-3′); Cd36_S (5′-TCCTCTGACATTTGCAGGTC-3′); Cd36_AS (5′-AAGGCATTGGCTGGAAGA-3′); Rps18_S (5′-CGCCATGTCTCTAGTGATCC-3′); Rps18_AS (5′-GGTCGATGTCTGCTTTCCTC-3′); Tbp_S (5′-ACTTCGTGCAAGAAATGCTGA-3′); and Tbp_AS (5′-TCTGGATTGTTCTTCACTCTTGG-3′).

### Immunoblotting.

Whole-cell lysates were prepared by lysing cells in RIPA buffer supplemented with 1× proteinase inhibitor cocktail. The protein concentration was quantified by BCA Protein Assays (Thermo Fisher Scientific). Membranes were blocked with 5% nonfat milk/TBST, probed with the primary antibodies anti-HIF2A (1:1,000), anti-HIF1A (1:1,000), anti–HA tag (1:10,000; Thermo Fisher Scientific; 26183), and anti–α-tubulin (1:5,000; MilliporeSigma; T6199), incubated with ECL reagents (Santa Cruz Biotechnology), and exposed to x-ray films.

### Luciferase assay.

A *Spry1* promoter region (–341 to +39 bp relative to the transcription start site [TSS]) and a *Pdk3* promoter region (–107 to +101 bp relative to the TSS) were PCR amplified from genomic DNA (*C57BL/6* male) and subcloned into pGL4.18 (Promega). pGL4.18_*Atp7a* has been reported previously (35). pGL4.18-Spry1, pGL4.18-Atp7a, and pGL4.18-Pdk3 (200 ng each) were cotransfected with pcDNA3-HIF1ATM, pcDNA3-HIF2ATM, or a control empty pcDNA3 plasmid (200 ng) into C2C12 myoblasts. pcDNA-LacZ (100 ng) expressing β-gal was cotransfected as an internal transfection control. Forty-eight hours after transfection, myoblasts were lysed with Luciferase Cell Lysis Buffer (NEB), followed by a firefly luciferase/β-gal assay with homemade luciferase and β-gal solutions. The luciferase assay solution consisted of 0.11 M Trizma, pH7.8, 0.5 M MgCl_2_, 0.1 M ATP, and 10 mM luciferin. The β-gal solution consisted of 4 mg/ml *O*-nitrophenyl-β-D-galactoside, 60 mM Na_2_HPO_4_, 40 mM NaH_2_PO_4_, 10 mM KCl, and 1 mM MgSO_4_.

### Statistics.

All values in the figures represent the mean ± SEM and reflect 3 or more biological replicates. Statistical significance was determined with GraphPad Prism 6 (GraphPad Software) using a 2-sided Student’s *t* test. A *P* value of less than 0.05 was considered statistically significant.

### Study approval.

All animal studies were approved by the IACUC of the University of Georgia and performed in strict accordance with institutional guidelines.

See complete unedited blots in the supplemental material.

## Author contributions

HY conceived of the study. LX, AY, AMB, JAC, and HY designed the study methodology. LX, AY, ASN, AMB, JAC, and HY performed the experiments. HY wrote the original draft of the manuscript, and LX, AY, AMB, JAC, and HY wrote, reviewed, and edited the manuscript. HY handled funding acquisition. AMB, JAC, and HY were responsible for resources. HY supervised the study.

## Supplementary Material

Supplemental data

## Version 1 03/13/2018

In-Press Preview

## Version 2 04/30/2018

Electronic publication

## Version 3 06/01/2018

Print issue publication

## References

[1] Goodell MA, Rando TA (2015). Stem cells and healthy aging. Science.

[2] Yin H, Price F, Rudnicki MA (2013). Satellite cells and the muscle stem cell niche. Physiol Rev.

[3] Murphy MM, Lawson JA, Mathew SJ, Hutcheson DA, Kardon G (2011). Satellite cells, connective tissue fibroblasts and their interactions are crucial for muscle regeneration. Development.

[4] Seale P, Sabourin LA, Girgis-Gabardo A, Mansouri A, Gruss P, Rudnicki MA (2000). Pax7 is required for the specification of myogenic satellite cells. Cell.

[5] Cheung TH (2012). Maintenance of muscle stem-cell quiescence by microRNA-489. Nature.

[6] Crist CG, Montarras D, Buckingham M (2012). Muscle satellite cells are primed for myogenesis but maintain quiescence with sequestration of Myf5 mRNA targeted by microRNA-31 in mRNP granules. Cell Stem Cell.

[7] Hausburg MA (2015). Post-transcriptional regulation of satellite cell quiescence by TTP-mediated mRNA decay. Elife.

[8] Ryall JG (2015). The NAD(+)-dependent SIRT1 deacetylase translates a metabolic switch into regulatory epigenetics in skeletal muscle stem cells. Cell Stem Cell.

[9] Rodgers JT (2014). mTORC1 controls the adaptive transition of quiescent stem cells from G0 to G(Alert). Nature.

[10] Jones NC (2005). The p38alpha/beta MAPK functions as a molecular switch to activate the quiescent satellite cell. J Cell Biol.

[11] Troy A, Cadwallader AB, Fedorov Y, Tyner K, Tanaka KK, Olwin BB (2012). Coordination of satellite cell activation and self-renewal by Par-complex-dependent asymmetric activation of p38α/β MAPK. Cell Stem Cell.

[12] Abou-Khalil R (2009). Autocrine and paracrine angiopoietin 1/Tie-2 signaling promotes muscle satellite cell self-renewal. Cell Stem Cell.

[13] Shea KL (2010). Sprouty1 regulates reversible quiescence of a self-renewing adult muscle stem cell pool during regeneration. Cell Stem Cell.

[14] Semenza GL (2012). Hypoxia-inducible factors in physiology and medicine. Cell.

[15] Mathieu J (2014). Hypoxia-inducible factors have distinct and stage-specific roles during reprogramming of human cells to pluripotency. Cell Stem Cell.

[16] Mohyeldin A, Garzón-Muvdi T, Quiñones-Hinojosa A (2010). Oxygen in stem cell biology: a critical component of the stem cell niche. Cell Stem Cell.

[17] Chaillou T, Lanner JT (2016). Regulation of myogenesis and skeletal muscle regeneration: effects of oxygen levels on satellite cell activity. FASEB J.

[18] Majmundar AJ (2015). HIF modulation of Wnt signaling regulates skeletal myogenesis in vivo. Development.

[19] Yang X, Yang S, Wang C, Kuang S (2017). The hypoxia-inducible factors HIF1α and HIF2α are dispensable for embryonic muscle development but essential for postnatal muscle regeneration. J Biol Chem.

[20] Brahimi-Horn MC, Pouysségur J (2007). Oxygen, a source of life and stress. FEBS Lett.

[21] Chou SC, Azuma Y, Varia MA, Raleigh JA (2004). Evidence that involucrin, a marker for differentiation, is oxygen regulated in human squamous cell carcinomas. Br J Cancer.

[22] Deal RB, Henikoff S (2010). A simple method for gene expression and chromatin profiling of individual cell types within a tissue. Dev Cell.

[23] Beerthuizen GI, Goris RJ, Kreuzer FJ (1989). Skeletal muscle Po2 during imminent shock. Arch Emerg Med.

[24] Richardson RS, Duteil S, Wary C, Wray DW, Hoff J, Carlier PG (2006). Human skeletal muscle intracellular oxygenation: the impact of ambient oxygen availability. J Physiol (Lond).

[25] Yin H (2013). MicroRNA-133 controls brown adipose determination in skeletal muscle satellite cells by targeting Prdm16. Cell Metab.

[26] Ren H, Accili D, Duan C (2010). Hypoxia converts the myogenic action of insulin-like growth factors into mitogenic action by differentially regulating multiple signaling pathways. Proc Natl Acad Sci USA.

[27] Zammit PS, Golding JP, Nagata Y, Hudon V, Partridge TA, Beauchamp JR (2004). Muscle satellite cells adopt divergent fates: a mechanism for self-renewal?. J Cell Biol.

[28] Menrad H (2010). Roles of hypoxia-inducible factor-1alpha (HIF-1alpha) versus HIF-2alpha in the survival of hepatocellular tumor spheroids. Hepatology.

[29] Liu W (2012). Hypoxia promotes satellite cell self-renewal and enhances the efficiency of myoblast transplantation. Development.

[30] Majmundar AJ (2012). O(2) regulates skeletal muscle progenitor differentiation through phosphatidylinositol 3-kinase/AKT signaling. Mol Cell Biol.

[31] Hu CJ, Sataur A, Wang L, Chen H, Simon MC (2007). The N-terminal transactivation domain confers target gene specificity of hypoxia-inducible factors HIF-1alpha and HIF-2alpha. Mol Biol Cell.

[32] Hardy D (2016). Comparative Study of Injury Models for Studying Muscle Regeneration in Mice. PLoS ONE.

[33] Scheuermann TH (2013). Allosteric inhibition of hypoxia inducible factor-2 with small molecules. Nat Chem Biol.

[34] Chakkalakal JV, Jones KM, Basson MA, Brack AS (2012). The aged niche disrupts muscle stem cell quiescence. Nature.

[35] Xie L, Collins JF (2011). Transcriptional regulation of the Menkes copper ATPase (Atp7a) gene by hypoxia-inducible factor (HIF2{alpha}) in intestinal epithelial cells. Am J Physiol, Cell Physiol.

[36] Lu CW, Lin SC, Chen KF, Lai YY, Tsai SJ (2008). Induction of pyruvate dehydrogenase kinase-3 by hypoxia-inducible factor-1 promotes metabolic switch and drug resistance. J Biol Chem.

[37] Xie L (2014). Hypoxia-inducible factor/MAZ-dependent induction of caveolin-1 regulates colon permeability through suppression of occludin, leading to hypoxia-induced inflammation. Mol Cell Biol.

[38] Yamaguchi M (2015). Calcitonin Receptor Signaling Inhibits Muscle Stem Cells from Escaping the Quiescent State and the Niche. Cell Rep.

[39] Martin SK (2010). Hypoxia-inducible factor-2 is a novel regulator of aberrant CXCL12 expression in multiple myeloma plasma cells. Haematologica.

[40] Hu CJ, Wang LY, Chodosh LA, Keith B, Simon MC (2003). Differential roles of hypoxia-inducible factor 1alpha (HIF-1alpha) and HIF-2alpha in hypoxic gene regulation. Mol Cell Biol.

[41] Das B (2012). HIF-2α suppresses p53 to enhance the stemness and regenerative potential of human embryonic stem cells. Stem Cells.

[42] Parmar K, Mauch P, Vergilio JA, Sackstein R, Down JD (2007). Distribution of hematopoietic stem cells in the bone marrow according to regional hypoxia. Proc Natl Acad Sci U S A.

[43] Kizaka-Kondoh S, Konse-Nagasawa H (2009). Significance of nitroimidazole compounds and hypoxia-inducible factor-1 for imaging tumor hypoxia. Cancer Sci.

[44] Latil M (2012). Skeletal muscle stem cells adopt a dormant cell state post mortem and retain regenerative capacity. Nat Commun.

[45] Boekstegers P, Riessen R, Seyde W (1990). Oxygen partial pressure distribution within skeletal muscle: indicator of whole body oxygen delivery in patients?. Adv Exp Med Biol.

[46] Ikossi DG (2006). Continuous muscle tissue oxygenation in critically injured patients: a prospective observational study. J Trauma.

[47] Christov C (2007). Muscle satellite cells and endothelial cells: close neighbors and privileged partners. Mol Biol Cell.

[48] Nombela-Arrieta C (2013). Quantitative imaging of haematopoietic stem and progenitor cell localization and hypoxic status in the bone marrow microenvironment. Nat Cell Biol.

[49] Spencer JA (2014). Direct measurement of local oxygen concentration in the bone marrow of live animals. Nature.

[50] Keith B, Johnson RS, Simon MC (2011). HIF1α and HIF2α: sibling rivalry in hypoxic tumour growth and progression. Nat Rev Cancer.

[51] Xie H, Simon MC (2017). Oxygen availability and metabolic reprogramming in cancer. J Biol Chem.

[52] Nakazawa MS, Keith B, Simon MC (2016). Oxygen availability and metabolic adaptations. Nat Rev Cancer.

[53] Holmquist-Mengelbier L (2006). Recruitment of HIF-1alpha and HIF-2alpha to common target genes is differentially regulated in neuroblastoma: HIF-2alpha promotes an aggressive phenotype. Cancer Cell.

[54] Koh MY, Lemos R, Liu X, Powis G (2011). The hypoxia-associated factor switches cells from HIF-1α- to HIF-2α-dependent signaling promoting stem cell characteristics, aggressive tumor growth and invasion. Cancer Res.

[55] Nakazawa MS (2016). Epigenetic re-expression of HIF-2α suppresses soft tissue sarcoma growth. Nat Commun.

[56] Zismanov V (2016). Phosphorylation of eIF2α is a translational control mechanism regulating muscle stem cell quiescence and self-renewal. Cell Stem Cell.

[57] Mohlin S (2015). PI3K-mTORC2 but not PI3K-mTORC1 regulates transcription of HIF2A/EPAS1 and vascularization in neuroblastoma. Cancer Res.

[58] Toschi A, Lee E, Gadir N, Ohh M, Foster DA (2008). Differential dependence of hypoxia-inducible factors 1 alpha and 2 alpha on mTORC1 and mTORC2. J Biol Chem.

[59] Briggs D, Morgan JE (2013). Recent progress in satellite cell/myoblast engraftment -- relevance for therapy. FEBS J.

[60] Ono Y, Masuda S, Nam HS, Benezra R, Miyagoe-Suzuki Y, Takeda S (2012). Slow-dividing satellite cells retain long-term self-renewal ability in adult muscle. J Cell Sci.

[61] Rocheteau P, Gayraud-Morel B, Siegl-Cachedenier I, Blasco MA, Tajbakhsh S (2012). A subpopulation of adult skeletal muscle stem cells retains all template DNA strands after cell division. Cell.

[62] Kuang S, Kuroda K, Le Grand F, Rudnicki MA (2007). Asymmetric self-renewal and commitment of satellite stem cells in muscle. Cell.

[63] Chen Z, Liu X, Mei Z, Wang Z, Xiao W (2014). EAF2 suppresses hypoxia-induced factor 1α transcriptional activity by disrupting its interaction with coactivator CBP/p300. Mol Cell Biol.

[64] Pawlus MR, Wang L, Hu CJ (2014). STAT3 and HIF1α cooperatively activate HIF1 target genes in MDA-MB-231 and RCC4 cells. Oncogene.

[65] Rasbach KA (2010). PGC-1alpha regulates a HIF2alpha-dependent switch in skeletal muscle fiber types. Proc Natl Acad Sci U S A.

[66] Call JA, Eckhoff MD, Baltgalvis KA, Warren GL, Lowe DA (2011). Adaptive strength gains in dystrophic muscle exposed to repeated bouts of eccentric contraction. J Appl Physiol.

[67] Pasut A, Jones AE, Rudnicki MA (2013). Isolation and culture of individual myofibers and their satellite cells from adult skeletal muscle. J Vis Exp.

[68] Motohashi N, Asakura Y, Asakura A (2014). Isolation, culture, and transplantation of muscle satellite cells. J Vis Exp.

[69] Gage GJ, Kipke DR, Shain W (2012). Whole animal perfusion fixation for rodents. J Vis Exp.

