# Supplementary material for: Transient HIF2A inhibition promotes satellite cell proliferation and muscle regeneration
Source: J Clin Invest. 2018 Apr 30;128(6):2339–55. doi: 10.1172/JCI96208 (PMC5983316; doi:10.1172/JCI96208)

SUPPLEMENTARY FIGURES AND LEGENDS

Supplementary Fig. 1

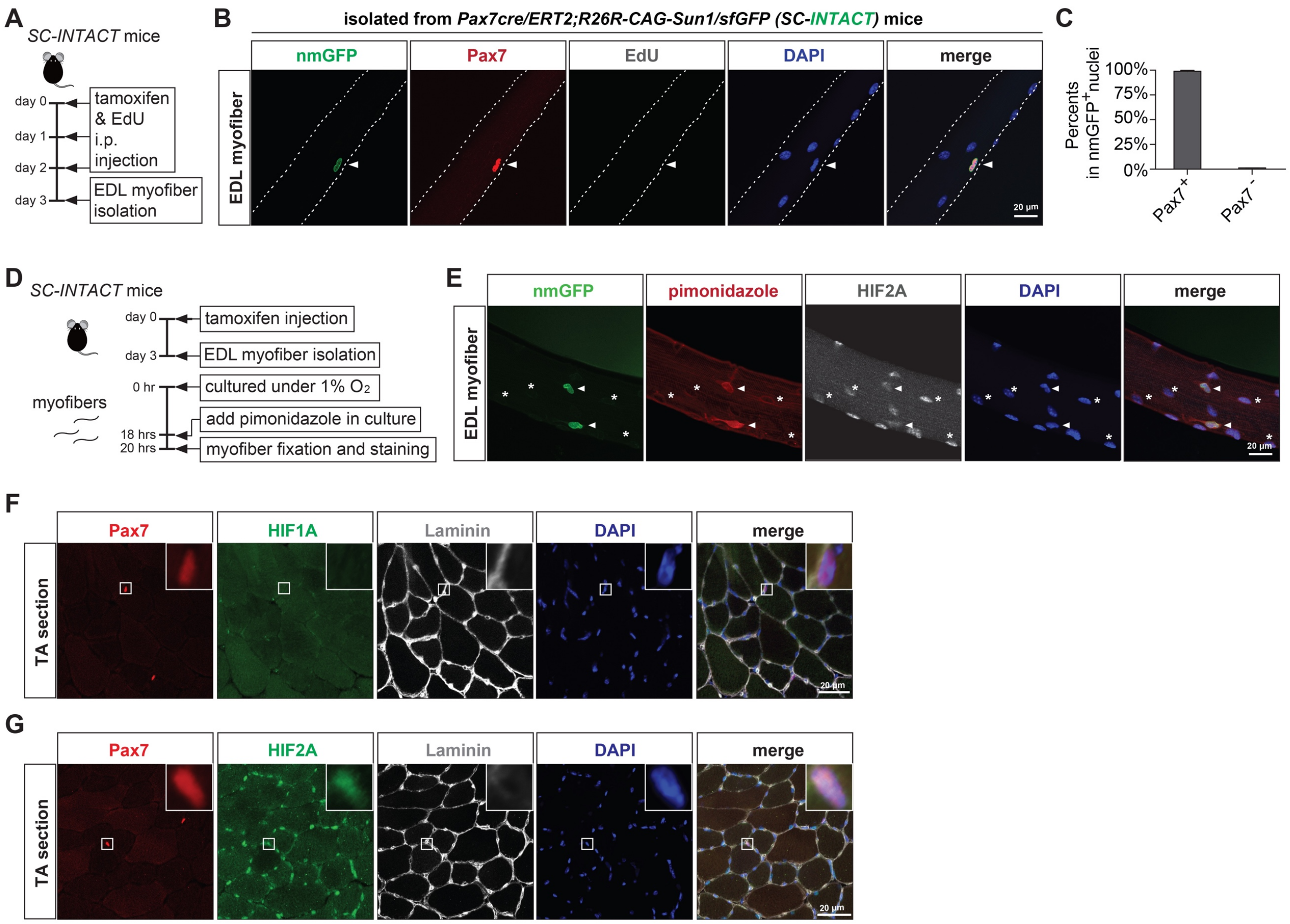

Supplementary Figure 1. Genetic tagging and labeling of satelite cell nuclear membrane and pimonidazole labeling of single isolated myofibers cultured under hypoxia.

(A) A diagram showing the genetic tagging and labeling of the nuclear membrane of quiescent SCs.

(B) Representative confocal images of uninjured EDL myofibers isolated from tamoxifen injected *Pax7cre/ERT2;R26R-CAG1-Sun1/sfGFP* (*SC-INTACT*) mice (n>50 myofibers from n=5 mice; after 3 consecutive days of tamoxifen and EdU injection). Immunofluorescence of Pax7, EdU and DAPI indicate that tamoxifen-induced expression of Sun1/sfGFP fusion protein specifically localizes to the nuclear membrane of satellite cells, which is cell cycle independent (labeled SCs are EdU^{-neg}). Arrowhead: satellite cell.

(C) Percents of Pax7^{+pos} nuclei (SC nuclei) and Pax7^{-neg} nuclei (myonuclei) in total nmGFP^{+pos} nuclei on uninjured EDL myofibers.

(D) A diagram summarizing the experimental procedure of pimonidazole labeling of single isolated myofibers cultured under hypoxia (1% O_2) for 20 hrs. Pimonidazole was added to the myofiber culture for the last 1.5 hrs to mimic the condition of in vivo pimonidazole labeling.

(E) Representative confocal images of EDL myofibers that were isolated from tamoxifen injected *SC-INTACT* mice (n>50 myofibers from n=3 mice), cultured under hypoxia, labeled with pimonidazole (50 μM). Fluorescent imaging reveals that nmGFP^{+pos} SCs (arrowheads) are hypoxic (pimonidazole^{+pos}) whereas myonuclei (asterisks) are pimonidazole^{-neg}.

(F-G) Representative cross-sectional images of uninjured TA muscles from *C57BL/6* mice (n=3 mice) with immunofluorescence of Pax7, HIF1A (F) or HIF2A (G), Laminin B2 and DAPI. Insets: zoomed-in images of quiescent SCs.

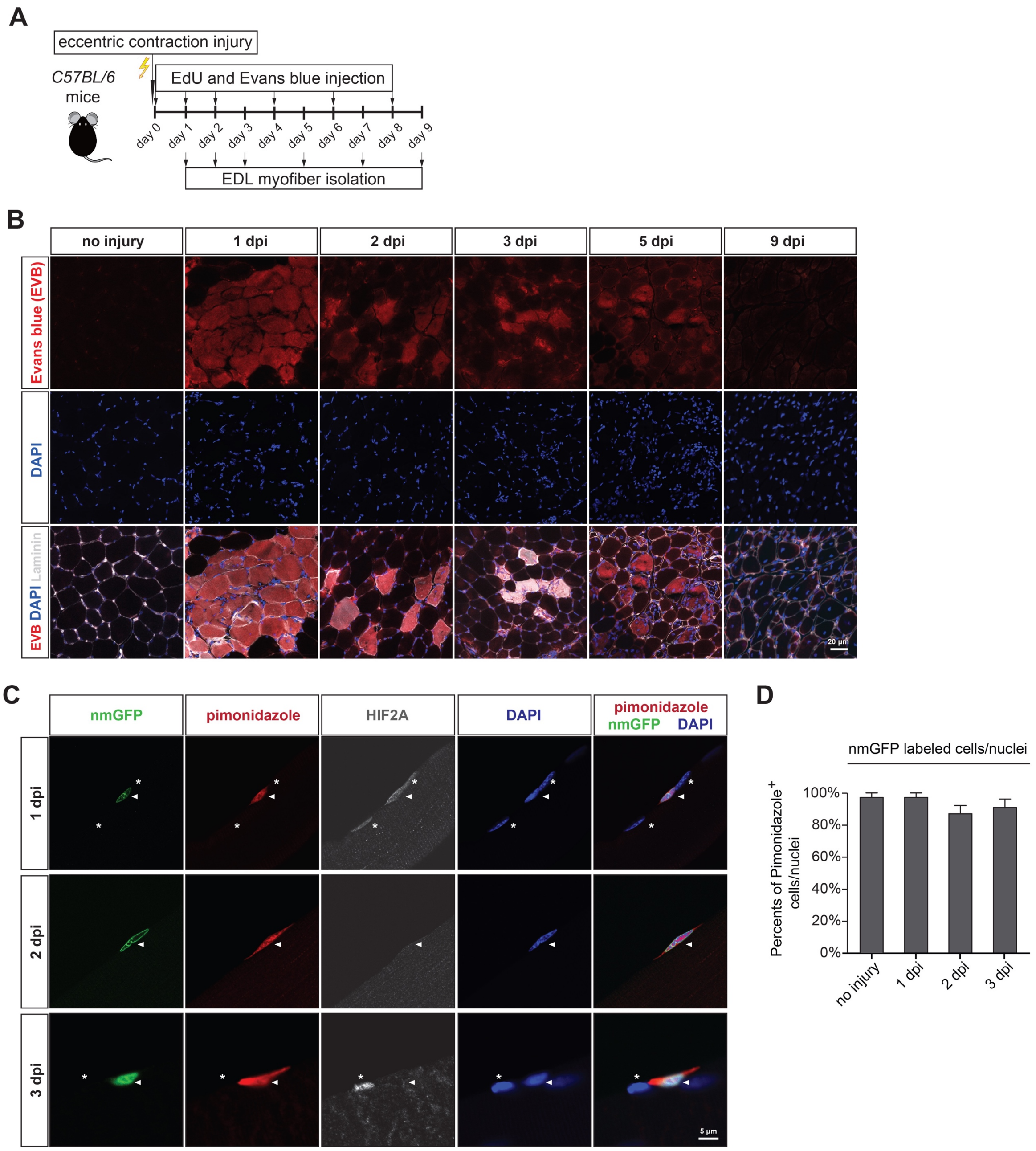

Supplementary Figure 2. Satellite cells maintain the hypoxic state when HIF2A expression diminishes upon eccentric contraction-induced injury.

(A) A timeline of characterizing SC dynamics after eccentric contraction-induced muscle injury. For each time point, EdU and Evans blue were injected 24 hrs before myofiber isolation.

(B) Representative cross-sectional images of uninjured and eccentric contraction-injured TA muscles (at 1, 2, 3, 4, 5, 9 dpi) from *C57BL/6* mice (n=3 mice/group) showing the fluorescence of Evans blue, Laminin B2 and DAPI.

(C) Representative images of EDL myofibers isolated from tamoxifen injected *SC-INTACT* mice at 1, 2 and 3 days post eccentric contraction-induced injury (n>50 myofibers; n=3 mice/time point). Pimonidazole was i.p. injected 1.5 hrs before euthanasia. nmGFP fluorescence and immunofluorescence of pimonidazole and HIF2A indicate that SCs maintain the pimonidazole^{+pos} hypoxic state from 1 dpi to 3 dpi, whereas HIF2A expression diminishes in hypoxic SCs at 2 dpi and 3 dpi. Arrowheads: SCs; asterisks: myonuclei.

(D) Percents of pimonidazole^{+pos} cells in nmGFP labeled cells on uninjured EDL myofibers and myofibers at 1-3 dpi.

Supplementary Fig. 3

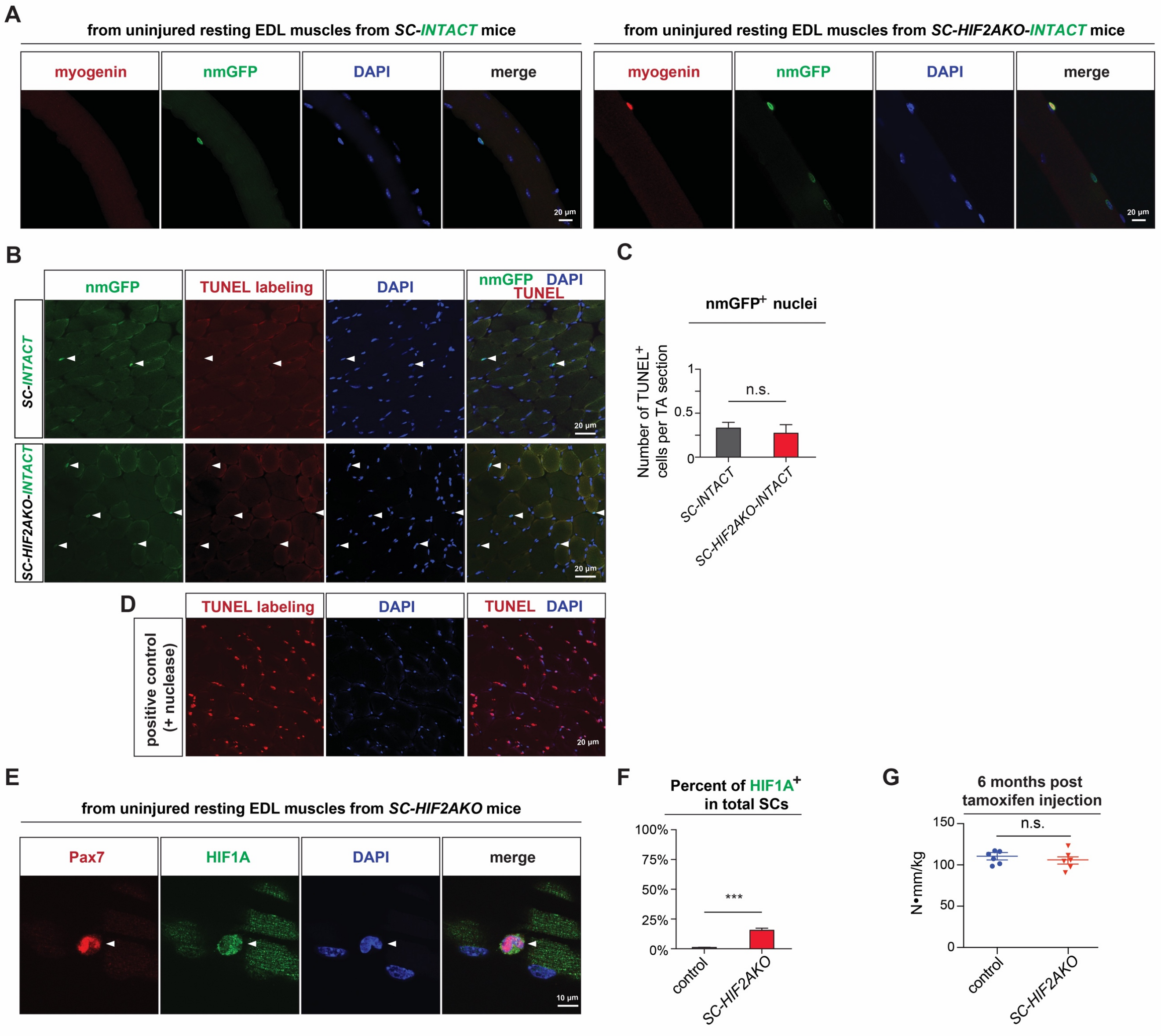

Supplementary Figure 3. SC-specific ablation of HIF2A leads to increased Myogenin and HIF1A expression, but does not results in SC apoptosis.

(A) Representative images of uninjured EDL myofibers isolated from tamoxifen injected *SC-HIF2AKO-INTACT* mice and control *SC-INTACT* mice (n>50 myofibers from n=3 mice/group; at 16 dpr). Fluorescence of nmGFP and Myogenin indicate that some nmGFP-labeled SCs are Myogenin^{+pos}.

(B) Representative cross-sectional images of uninjured TA muscles from tamoxifen injected *SC-HIF2AKO-INTACT* mice and control *SC-INTACT* mice (n=3 mice/group; at 16 dpr) showing

nmGFP^{+pos} cells (SC lineage; arrowheads) lack of the fluorescence of TUNEL labeling. Arrowheads: nmGFP^{+pos} cells.

(C) Percents of TUNEL^{+pos}/nmGFP^{+pos} cells in *SC-HIF2AKO-INTACT* mice and control *SC-INTACT* mice.

(D) Representative images of the technical positive control for TUNEL labeling showing pronounced fluorescence of TUNEL labeling after nuclease treatment.

(E) Representative images of uninjured EDL myofibers isolated from tamoxifen injected *SC-HIF2AKO* mice (n>50 myofibers from n=3 mice; at 16 dpr). Immunofluorescence of Pax7 and HIF1A indicate that some Pax7^{+pos} SCs are HIF1A^{+pos}. Arrowheads: SC.

(F) Percents of HIF1A^{+pos} SCs in *SC-HIF2AKO* mice and the control littermates.

(G) The maximal torque of uninjured TA muscles from *SC-HIF2AKO* mice and age-matched control littermates (n=6 mice/group) at 6 months after tamoxifen -nduced recombination.

Supplementary Fig. 4

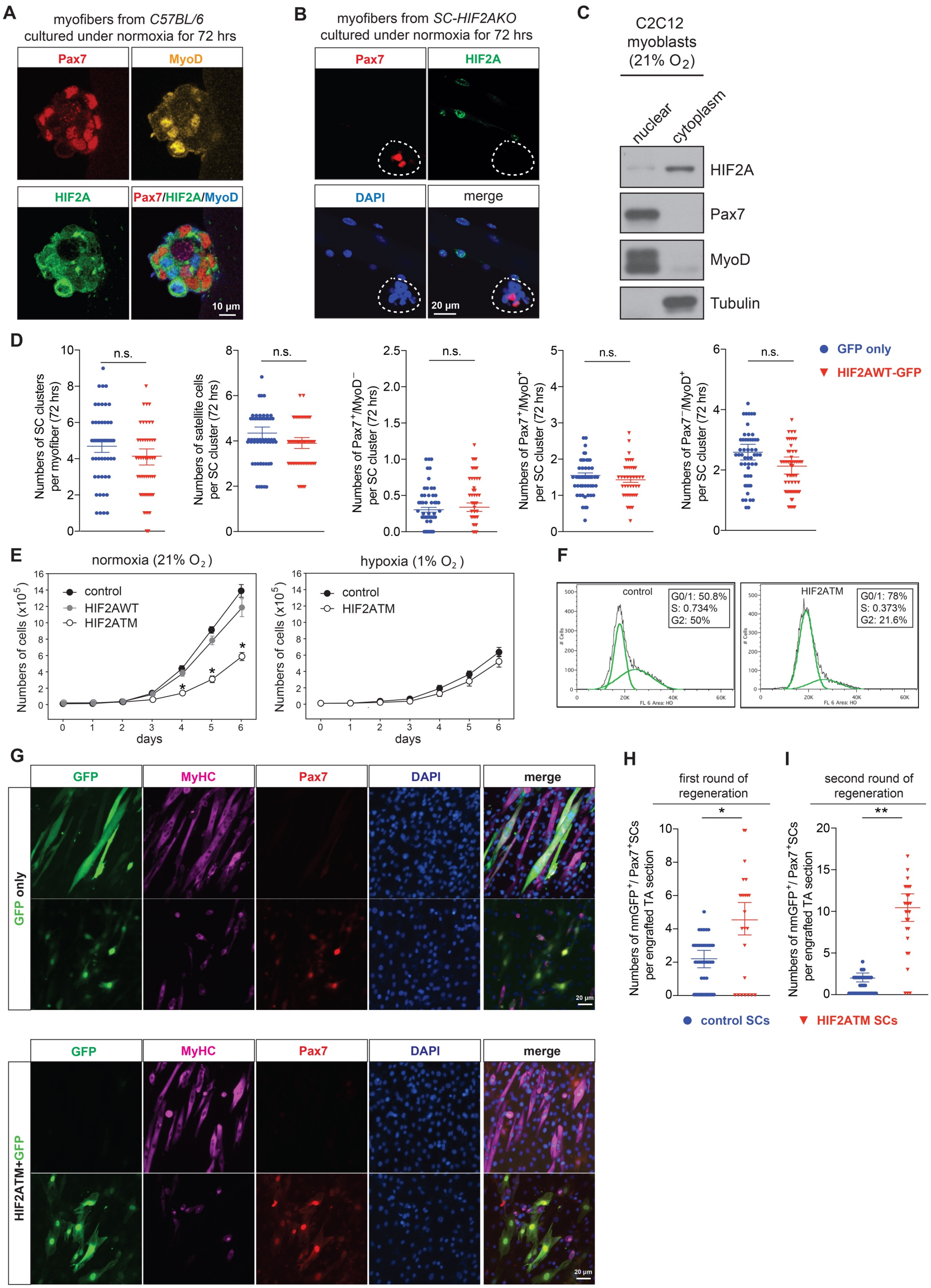

Supplementary Figure 4. HIF2A localizates in the cytoplasm of cultured SCs and C2C12 myoblasts under normoxia (21% pO_2) and effects of transfection of wildtype and O_2-insensitive HIF2A in primary and C2C12 myoblasts.

(A) Representative images of a SC cluster on myofibers isolated from *C57BL/6* mice (n>50 clusters from n=3 mice) and cultured for 72-hrs under normoxia showing immunofluorescence of Pax7, MyoD, HIF2A and DAPI. Notably, HIF2A immunofluorescence localizes mostly in the cytoplasm, which is distinct from the nuclear localization of Pax7 and MyoD.

(B) Representative images of a SC cluster on myofibers isolated from tamoxifen injected *SC-HIF2AKO* mice (n>50 clusters from n=3 mice; at 10 dpr) and cultured for 72-hrs under normoxia showing immunofluorescence of Pax7, HIF2A and DAPI.

(C) Immunoblotting of Pax7, MyoD, HIF2A and Tubulin in nuclear and cytoplasmic fractions of C2C12 myoblasts cultured under normoxia.

(D) From left to right, numbers of SC clusters, $Pax7^{+pos}$ SCs per cluster, $Pax7^{+pos}$/$MyoD^{-neg}$, $Pax7^{+pos}$/$MyoD^{+pos}$ and $Pax7^{-neg}$/$MyoD^{+pos}$ SCs per SC cluster (n>50 myofibers) after transfection of wildtype HIF2A in SCs cultured for 72-hrs under normoxia.

(E) Numbers of primary myoblasts after transfection of control, wildtype HIF2A (HIF2AWT) and O2-insensitive HIF2A (HIF2ATM) and cultured under normoxia or hypoxia (1% O_2) for 1-6 days ($1x10^4$ seeded at day 0; n=6 replicates).

(F) Histograms of Hoeschst 33258 intensity distribution in control and HIF2ATM transfected primary myoblasts and results of cell cycle analysis (FlowJo).

(G) Representative images of C2C12 myoblasts transfected with control or HIF2ATM plasmids and differentiated in 2% horse serum for 5 days. Non-transfected (GFP^{-neg}) and transfected (GFP^{+pos}) cells were immunostained for MyHC, Pax7 and DAPI.

(H, I) Numbers of $nmGFP^{+pos}$/$Pax7^{+pos}$ engrafted SCs per TA muscle section after the 1st round (at 21 dpi; n=5 mice/group; H) and the 2nd round of regeneration (at 30 dpi; n=6 mice/group; I).

Supplementary Fig. 5

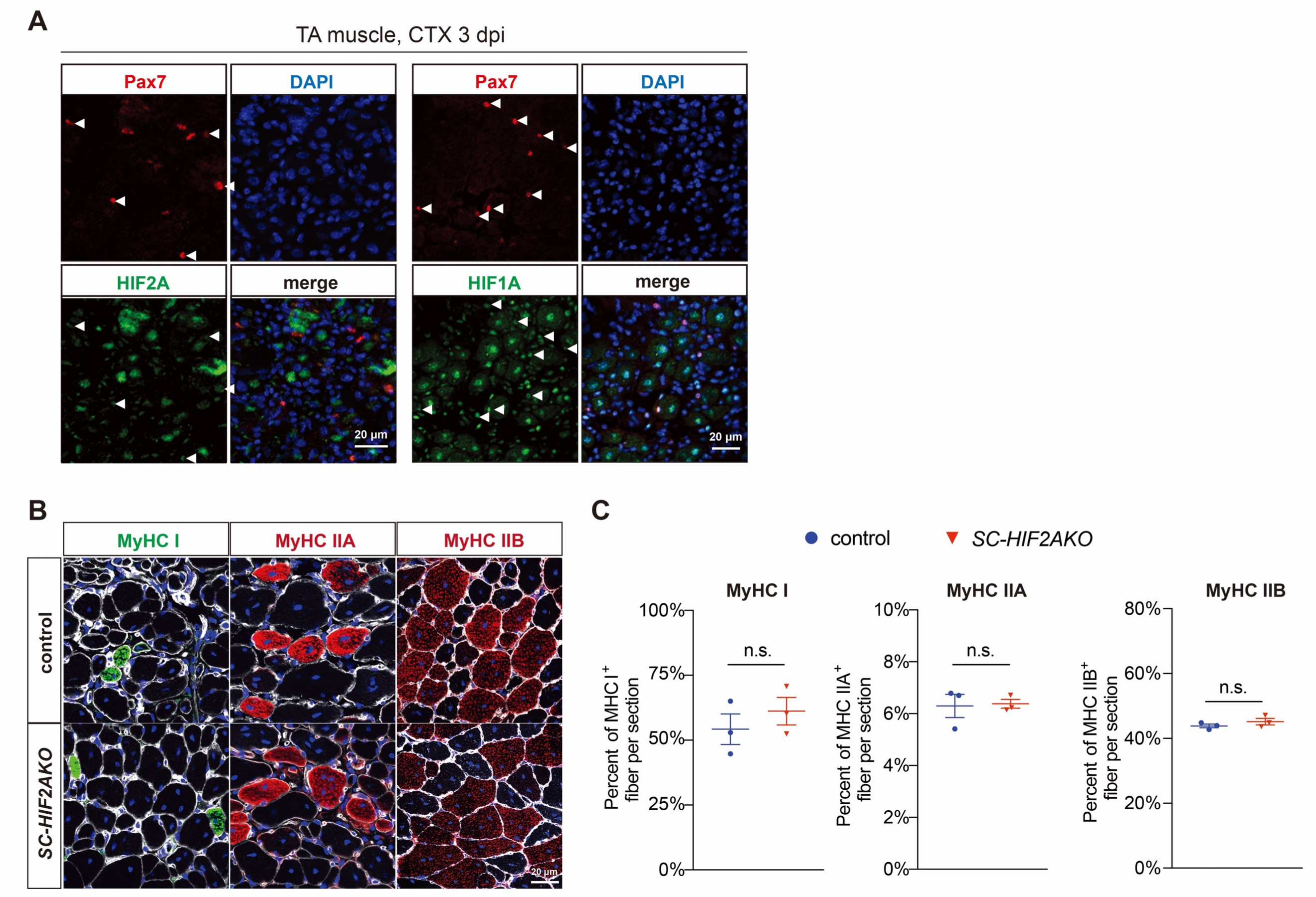

Supplementary Figure 5. HIF expression in activated satellite cells after CTX-induced injury and compositions of MyHC I, IIA and IIB-positive myofibers after muscle regeneration of *SC-HIF2AKO* mice.

(A) Representative cross-sectional images of CTX-injured TA muscles (at 3 dpi) from *C57BL/6* mice (n=6 mice/group) showing immunofluorescence of Pax7, HIF2A or HIF1A and DAPI. Arrowheads: SCs.

(B) Representative cross-sectional images of TA muscles (at 30 dpi) from *SC-HIF2AKO* mice and control littermates (n=3 mice/group) showing immunofluorescence of type I MyHC, type IIA MyHC or type IIB MyHC along with Laminin B2 and DAPI.

(C) The percents of myofibers positive for type I MyHC, type IIA MyHC or type IIB MyHC.

Supplementary Fig. 6

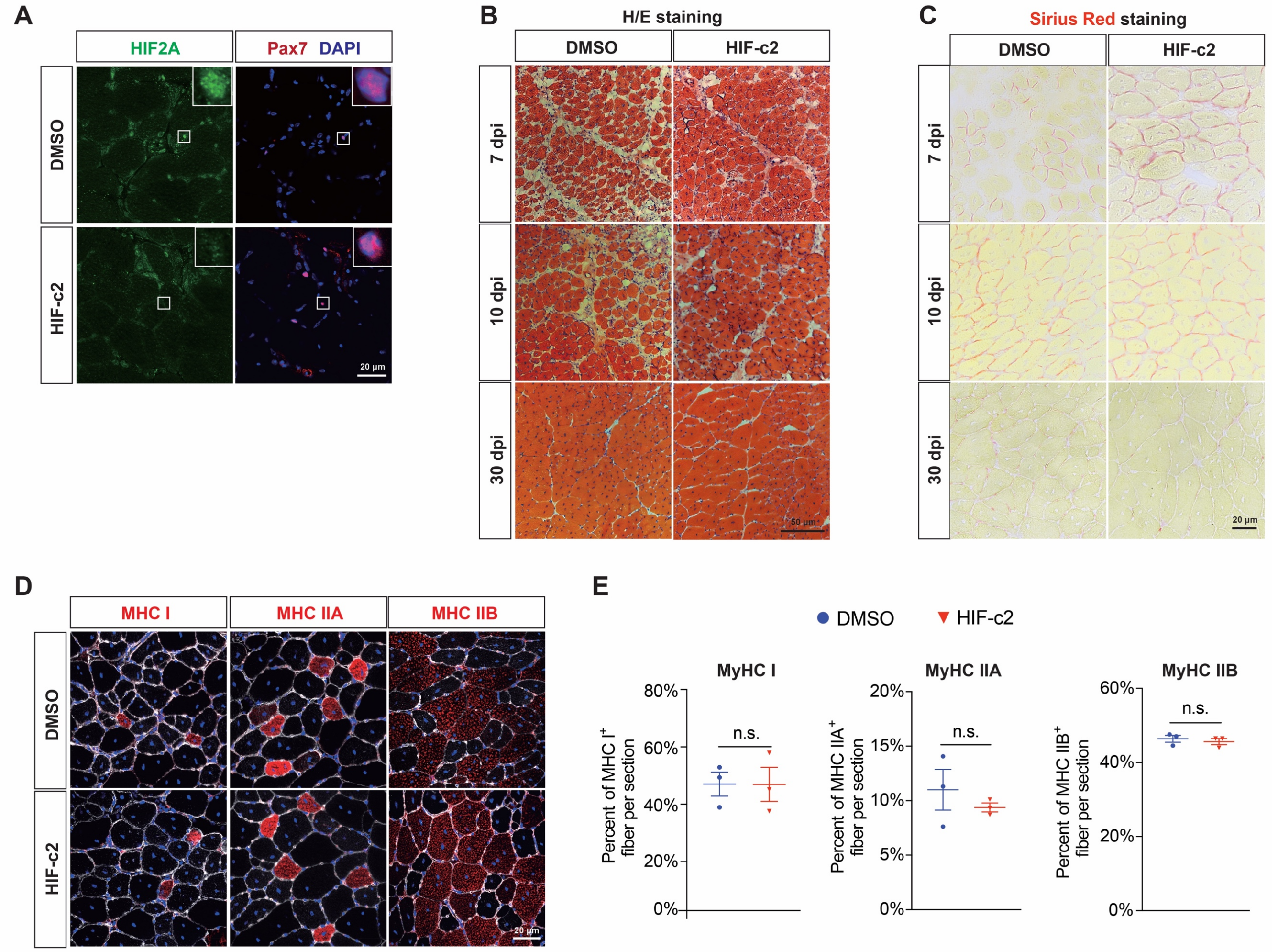

Supplementary Figure 6. Inhibition of HIF2A by HIF-c2, muscle morphology, collagen content and myofiber type composition of HIF-c2-treated muscle during or after regeneration.

(A) Representative cross-sectional images of 1% DMSO treated or HIF-c2 treated uninjured TA muscles from *C57BL/6* mice (n=3 mice/group) showing immunofluorescence of Pax7, HIF2A and DAPI. Insets are zoomed-in images of SCs.

(B) Representative images of H/E staining of CTX-injured TA muscle cross-sections (at 7, 10 and 30 dpi) from *C57BL/6* mice (n=3 mice/group) that were treated by 1% DMSO or HIF-c2.

(C) Representative images of Sirius Red staining (for collagen) of CTX-injured TA muscle cross-sections (at 7, 10 and 30 dpi) from *C57BL/6* mice (n=3 mice/group) that were treated by 1% DMSO or HIF-c2.

(D) Representative cross-sectional images of CTX-injured TA muscles (at 30 dpi) that were treated with 1% DMSO or HIF-c2, showing immunofluorescence of type I MyHC, type IIA MyHC or type IIB MyHC along with Laminin B2 and DAPI.

(E) The percents of myofibers positive for type I MyHC, type IIA MyHC or type IIB MyHC.

Supplementary Fig. 7

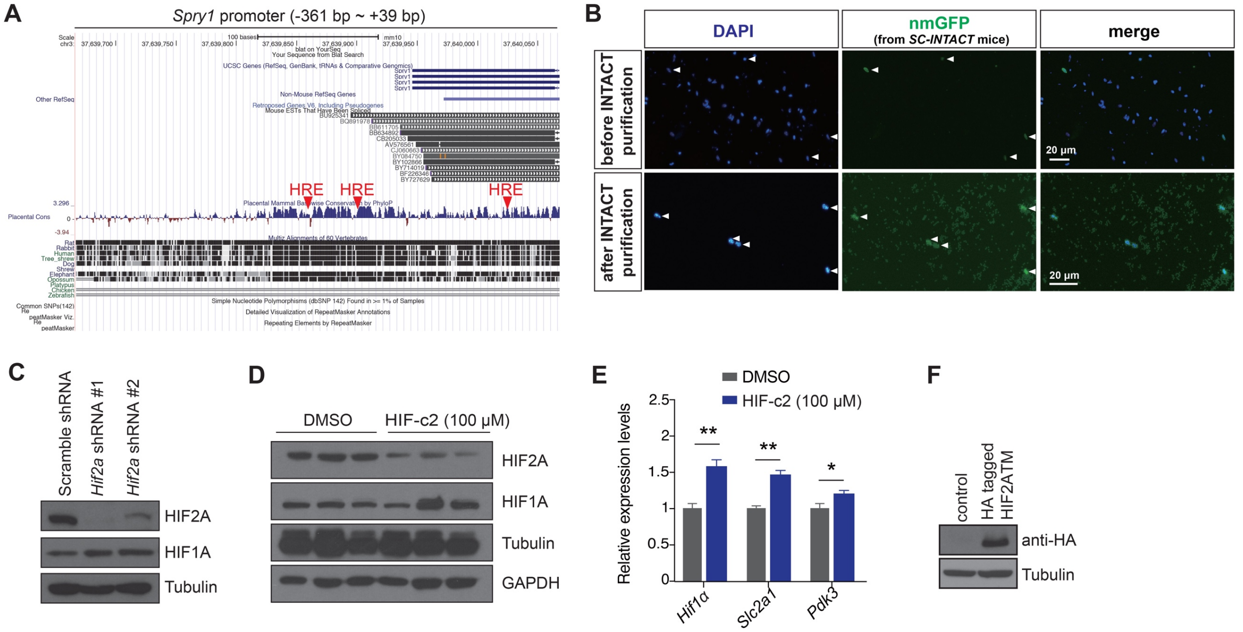

Supplementary Figure 7. HIF2A binds to HREs in *Spry1* promoter, isolation of nuclei tagged in specific cell types (INTACT) for SCs and reduced HIF2A expression in HIF2A shRNA expressing or HIF-c2 treated C2C12 myoblasts.

(A) A view from UCSC genome browser showing positions of three Hypoxia-Response Elements (HREs; red) in the *Spry1* proximal promoter (mouse genome).

(B) Representative images showing ratios of nmGFP^{+pos} SC nuclei and nmGFP^{-neg} nuclei (including myonuclei and nuclei from other types of cells in muscle) from *SC-INTACT* mice before and after INTACT purification with magnetic Dynabeads. Notably, nmGFP^{+pos} SC nuclei were affinity attached to Dynabeads (dots with weak green autofluorescence) by anti-GFP/anti-Myc antibodies and were enriched after purification.

(C) Immunoblotting of HIF2A, HIF1A and Tubulin in C2C12 myoblasts transduced with retrovirus expressing control or HIF2A shRNAs (#1 and #2).

(D) Immunoblotting of HIF2A, HIF1A, Tubulin and GAPDH in C2C12 myoblasts, which were treated with HIF-c2 or 1% DMSO (n=3 independent treatments/group).

(E) RT-qPCR assays reveal increased mRNA levels of *HIF1A* and *HIF1A* target genes, *Slc2a1/Glut1* and *Pdk3* in C2C12 myoblasts in response to HIF-c2 (100 µM) treatment.

(F) Immunoblotting (using an antibody against HA-tag) showing HA-tagged HIF2ATM is ectopically expressed in C2C12 myoblasts.

Full unedited blot for Figure 7F

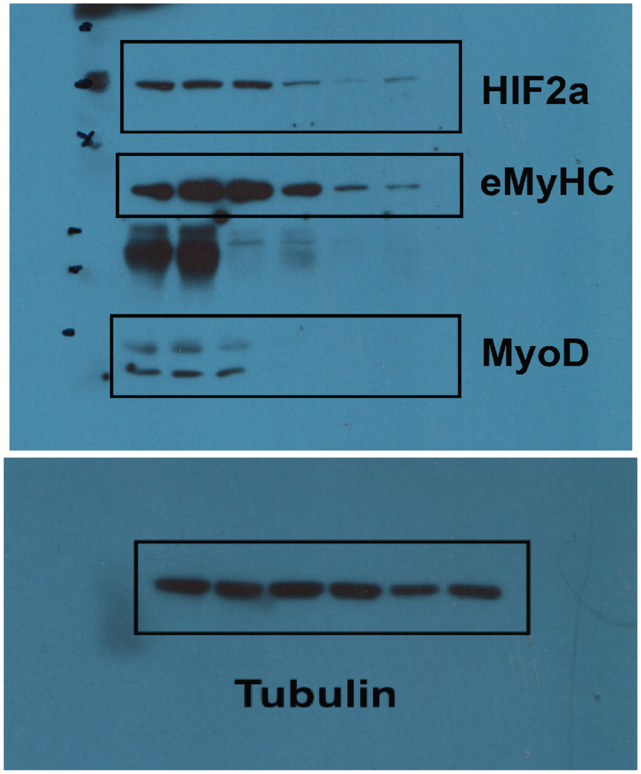

Full unedited blot for Figure S7C

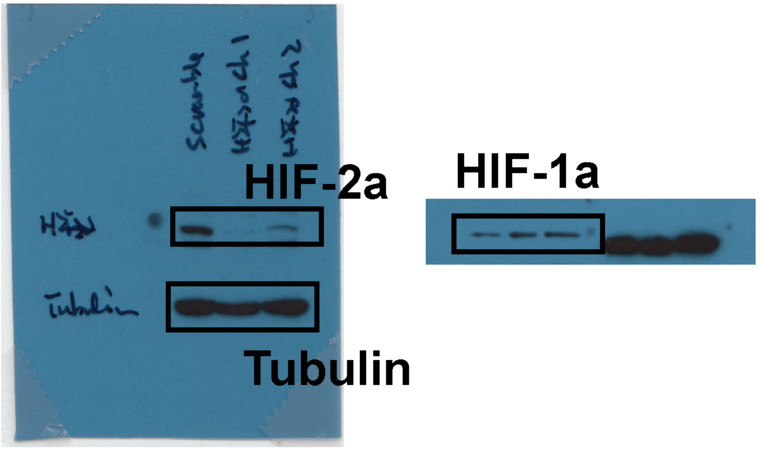

Full unedited blot for Figure S7D

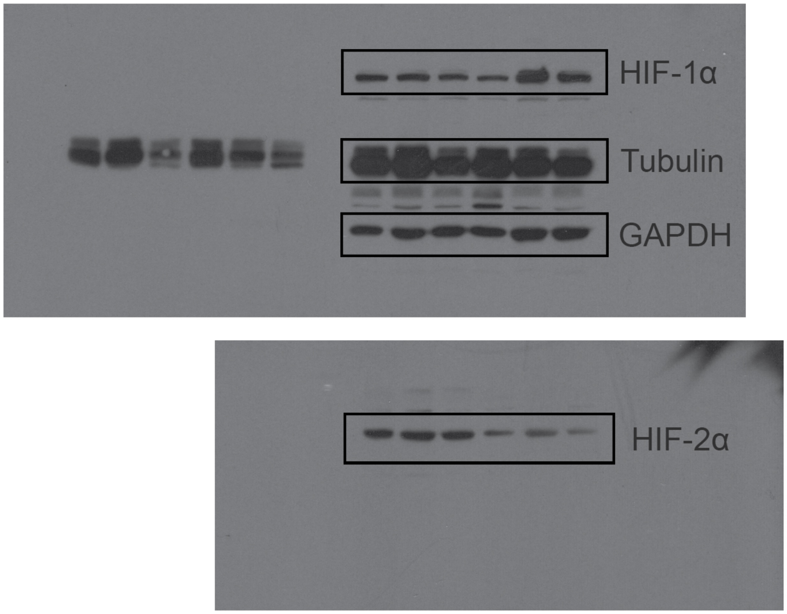

Full unedited blot for Figure S4C

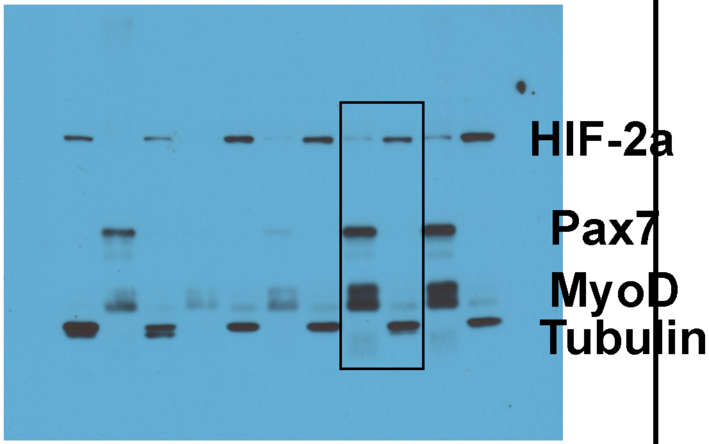

Full unedited blot for Figure S7F

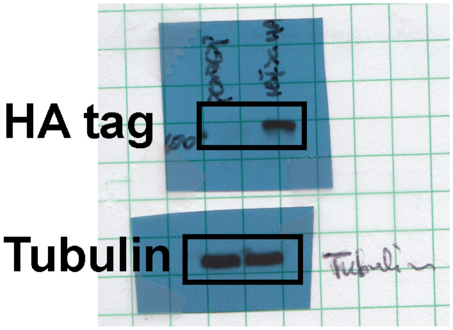

Supplement: Supplemental data [file jci-128-96208-s009.pdf]
